# Immediate birth for women between 34 and 37 weeks of gestation with prolonged preterm prelabour rupture of membranes and detection of vaginal or urine group B streptococcus: an economic evaluation

**DOI:** 10.1111/1471-0528.17119

**Published:** 2022-03-08

**Authors:** Jeremy Dietz, Jane Plumb, Philip Banfield, Aung Soe, Fadi Chehadah, Stacey Chang‐Douglass, Gabriel Rogers

**Affiliations:** ^1^ Centre for Guidelines National Institute for Health and Care Excellence (NICE) London UK; ^2^ Group B Strep Support Haywards Heath West Sussex UK; ^3^ Glan Clwyd Hospital Bodelwyddan Rhyl UK; ^4^ Oliver Fisher Neonatal Unit Medway Maritime Hospital Gillingham Kent UK; ^5^ Centre for Guidelines National Institute for Health and Care Excellence (NICE) Manchester UK; ^6^ Division of Population Health, Manchester Centre for Health Economics The University of Manchester Manchester UK

**Keywords:** antibiotics, caesarean delivery, economics of health care, group B streptococcus, health economists, infection, neonatal, paediatrics

## Abstract

**Objective:**

What are the costs, benefits and harms of immediate birth compared with expectant management in women with prolonged preterm prelabour rupture of membranes (PPROM) at 34^+0^–36^+6^ weeks of gestation and detection of vaginal or urine group B streptococcus (GBS)?

**Design:**

Mathematical decision model comprising three independent decision trees.

**Setting:**

UK National Health Service (NHS) and personal social services perspective.

**Population:**

Women testing positive for GBS with PPROM at 34^+0^–36^+6^ weeks of gestation.

**Methods:**

The model estimates lifetime costs and quality‐adjusted life years (QALYs) using evidence from randomised trials, UK NHS data sources and further observational studies. Simulated events include neonatal infections, morbidity associated with preterm birth and consequences of caesarean birth. Deterministic and probabilistic sensitivity analyses (PSAs) were performed.

**Main outcome measures:**

QALYs, costs and incremental cost‐effectiveness ratio (ICER).

**Results:**

In this population, immediate birth dominates expectant management: it is more effective (average lifetime QALYs, 24.705 versus 24.371) and it is cheaper (average lifetime costs, £14,372 versus £19,311). In one‐way sensitivity analysis, results are robust to all but the odds ratio estimating the relative effect on incidence of infections. Threshold analysis shows that the odds of infection only need to be >1.5% with expectant management for the benefit of avoiding infections to outweigh the disadvantages of immediate birth. In PSA, immediate birth is the preferred option in >80% of simulations.

**Conclusions:**

Neonatal GBS infections are expensive to treat and may result in substantial adverse health consequences. Therefore, immediate birth, which is associated with a reduced risk of neonatal infection compared with expectant management, is expected to generate better health outcomes and decreased lifetime costs.

**Tweetable abstract:**

For women with preterm prelabour rupture of membranes and group B streptococcus in vaginal or urine samples, immediate birth is associated with improved health in their babies and reduced costs, compared with expectant management.

## Tweetable abstract

For women with preterm prelabour rupture of membranes and group B streptococcus in vaginal or urine samples, immediate birth is associated with improved health in their babies and reduced costs, compared with expectant management.

## 1 INTRODUCTION

The incidence of preterm prelabour rupture of membranes (PPROM, defined as the rupture of membranes before the onset of labour at less than 37 completed weeks of gestation) ranges from 3% to 10% of pregnancies.^1^ For women with no additional risk factors, current professional guidelines recommend expectant management at any gestational age.^2^ This advice is based on multiple trials showing small but significant excess morbidity and mortality in babies randomised to immediate birth, defined as induction of labour or caesarean birth, even in the late‐preterm period (34^+0^–36^+6^ weeks of gestation).^3^

One relatively common risk factor that may alter the balance of benefits, harms and costs between expectant management and early birth is maternal carriage of group B streptococcus (GBS). Vertical transmission of GBS is the most common cause of early‐onset neonatal sepsis in the UK.^4^ The estimated prevalence of GBS in pregnant women ranges from 12% using vaginal or urine samples to 21% in a UK study that also included rectal swabs.^5^, ^6^ Therefore, it is plausible that the heightened risk of infection associated with a prolonged gap between rupture of membranes and birth could outweigh the harms of early birth. Current Royal College of Obstetricians and Gynaecologists (RCOG) guidance contains a weak recommendation that, in women testing positive for GBS with late‐preterm PPROM, it ‘may be beneficial to expedite delivery’;^7^ however, there has been no previous attempt to synthesise relevant evidence in a thoroughgoing decision analysis.

The objective of our analysis was to compare the benefits, harms and costs of immediate birth versus expectant management in women with prolonged PPROM at 34^+0^–36^+6^ weeks of gestation and vaginal or urine GBS detection.

## 2 METHODS

We undertook this analysis as part of an update to the National Institute for Health and Care Excellence (NICE) national guidance on the diagnosis and management of neonatal infection.^8^ We followed NICE methods and used the Consolidated Health Economic Evaluation Reporting Standards (CHEERS) statement^9^, ^10^ to report the analysis.

We built a decision model in microsoft excel (Microsoft, Redmond, WA, USA). The simulated population is women with PPROM at 34^+0^–36^+6^ weeks of gestation and vaginal or urine GBS detection. The model was a cost–utility analysis (CUA) comparing two approaches: immediate birth and expectant management. We measure outcomes in quality‐adjusted life years (QALYs) and costs in 2018/19 GBP (£). Our primary outcome is an incremental cost‐effectiveness ratio (ICER), expressed as cost per QALY gained. The model has a lifetime horizon, to reflect all important differences in costs and outcomes between the two approaches. Nevertheless, all relevant transitions in the model happen within 72 h of birth. The analysis adopts a UK National Health Service (NHS) and personal social services (PSS) perspective. Following standard UK practice, it discounts all costs and QALYs, applying a weight that is 3.5% lower for every year into the future that the outcomes occur.

**FIGURE 1:**
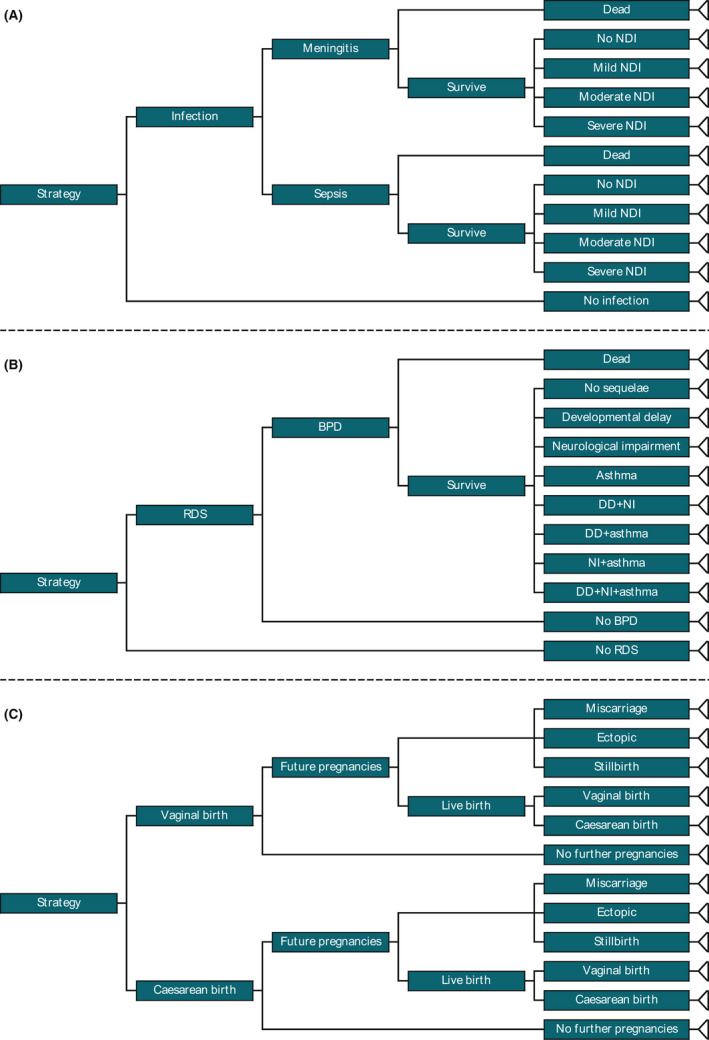
Structure of original cost‐utility model. BPD, bronchopulmonary dysplasia; DD, developmental delay; NDI, neurodevelopmental impairment; NI, neurological impairment; RDS, respiratory distress syndrome. The model comprises three independent decision trees, simulating: (A) infections and their sequelae; (B) RDS and its sequelae; and (C) mode of birth and its impact on subsequent pregnancies

The model comprises three independent decision trees (Figure 1). The first determines the risk of infection among babies. The model subdivides infections into meningitis and sepsis, both of which are associated with risks of long‐term disability or death. The second decision tree calculates the proportion of babies experiencing health effects of prematurity. As none of the available evidence reports long‐term neurodevelopmental outcomes, we use the short‐term outcome of respiratory distress syndrome (RDS) as a proxy measure. We then project long‐term sequelae, using evidence of lifelong health problems with which RDS is associated, via chronic lung disease (bronchopulmonary dysplasia) and its consequences. We do not assume this relationship is necessarily causal; rather, we use RDS rates as an indicator of the problems faced by late‐preterm babies, some of which have lasting consequences. The final decision tree simulates outcomes relating to the mode of birth. The model determines the likelihood of caesarean or vaginal birth, and then estimates the impact of caesarean birth on future pregnancies, including costs associated with future births (which are more likely to be caesarean deliveries if the index birth was a caesarean), and costs and QALY loss as a result of adverse pregnancy outcomes (using evidence that risks of ectopic pregnancy, miscarriage and stillbirth are increased in women with a history of caesarean birth).^11^ Finally, we sum the expected costs and QALYs from the three decision trees to estimate the net consequences of each strategy.

### 2.1 Parameters

Table 1 summarises the input parameters for the models; Appendices S1–S4 provide full details of their derivation.

**TABLE 1:** Key inputs used in the model

Parameter	Value (95% CI)	Probabilistic parameters	Source
Probabilities			
Neonatal infection			
Baseline probability – expectant	0.152 (0.065, 0.268)	Beta: α = 7; β = 39	12
Odds ratio: expectant vs immediate	2.927 (0.327, 26.190)	Lognormal: μ = 1.07; σ = 1.12	See Appendix S2
Probability – immediate	0.058		
Respiratory distress syndrome			
Baseline odds – immediate	0.087 (0.071, 0.106)	Lognormal: μ = −2.45; σ = 0.10	See Appendix S1
Odds ratio: expectant vs immediate	0.675 (0.503, 0.904)	Lognormal: μ = −0.39; σ = 0.15	See Appendix S2
Probability – immediate	0.080		
Probability – expectant	0.055		
Caesarean birth			
Baseline probability – expectant	0.301 (0.300, 0.302)	Beta: α = 179 475; β = 416 626	18
Odds ratio: expectant vs immediate	0.776 (0.644, 0.936)	Lognormal: μ = −0.25; σ = 0.10	See Appendix S2
Probability – immediate	0.357		
Probability of meningitis given infection	0.110 (0.085, 0.139)	Beta: α = 57; β = 460	36
Probability of death given meningitis			
32–36 weeks of gestation	0.093 (0.027, 0.195)	Beta: α = 4; β = 39	37
37+ weeks of gestation	0.043 (0.021, 0.072)	Beta: α = 10; β = 225	
Fitted probability – immediate	0.091		
Fitted probability – expectant	0.083		
Probability of death given sepsis			
34–36 weeks of gestation	0.061 (0.013, 0.143)	Beta: α = 3; β = 46	36
37+ weeks of gestation	0.027 (0.013, 0.047)	Beta: α = 9; β = 321	
Fitted probability – immediate	0.060		
Fitted probability – expectant	0.054		
Neurodevelopmental sequelae of meningitis			
None	0.614 (0.535, 0.692)	Dirichlet: α_1_ = 90.10; α_2_ = 28.76; α_3_ = 18.93; α_4_ = 8.95	30
Mild	0.196 (0.136, 0.264)		
Moderate	0.129 (0.081, 0.187)		
Severe	0.061 (0.029, 0.104)		
Neurodevelopmental sequelae of sepsis			
None	0.746 (0.641, 0.838)	Dirichlet: α_1_ = 55.22; α_2_ = 3.33; α_3_ = 10.29; α_4_ = 5.18	30
Mild	0.045 (0.011, 0.100)		
Moderate	0.139 (0.072, 0.222)		
Severe	0.070 (0.023, 0.138)		
Probability of BPD given RDS			
Baseline probability (31 weeks)	0.361 (0.341, 0.381)	Beta: α = 806; β = 1427	38
OR per week of gestational age	0.620 (0.600, 0.640)	Lognormal: μ = −0.48; σ = 0.02	
Fitted probability – immediate	0.080		
Fitted probability – expectant	0.064		
Probability of sequelae given BPD			
Death	0.017 (0.000, 0.061)	Beta: α = 1; β = 59	39
Developmental delay	0.343 (0.197, 0.505)	Beta: α = 12; β = 23	
Neurological impairment	0.143 (0.050, 0.275)	Beta: α = 5; β = 30	
Asthma	0.359 (0.218, 0.514)	Beta: α = 14; β = 25	
Hazard ratio for death associated with NDI			
Mild	1.0	–	24
Moderate	1.5 (0.7, 3.2)	Lognormal: μ = 0.41; σ = 0.39	
Severe	6.2 (3.3, 11.8)	Lognormal: μ = 1.83; σ = 0.33	
Quality of life (absolute disutility)			
Mild neurodevelopmental impairment	0.179 (0.105, 0.268)	Beta: α = 14.73; β = 67.58	19
Moderate neurodevelopmental impairment	0.298 (0.196, 0.411)	Beta: α = 20.31; β = 47.85	
Severe neurodevelopmental impairment	0.558 (0.391, 0.718)	Beta: α = 18.95; β = 15.01	
Asthma	0.058 (0.053, 0.063)	Beta: α = 486.9; β = 7907.4	21
Costs			
Antenatal costs – immediate	£797.75	See Appendix S4	5, 40
Antenatal costs – expectant	£2455.30		
Delivery costs – immediate	£3250.58	See Appendix S4	40
Delivery costs – expectant	£3117.76		
Postnatal care costs			
Expected costs if no infections – immediate	£4942.16	See Appendix S4	5, 40, 41
Expected costs if no infections – expectant	£4009.61		
Additional costs per infection	£7368.72	See Appendix S4	40, 41

Abbreviations: BPD, bronchopulmonary dysplasia; NDI, neurodevelopmental impairment; RDS, respiratory distress syndrome.

#### 2.1.1 Effectiveness

The estimates we use to capture the effects of the strategies come from systematic reviews of relevant randomised controlled trials (RCTs).^3^, ^8^ For the probability of infection, it is critical to use evidence that reflects mothers who test positive for GBS only. Therefore, we use the odds ratio (OR) synthesised from the GBS‐positive subgroups of two RCTs applied to a baseline probability of infection.^12^, ^13^ We assume the only impact that GBS status has on RDS and caesarean birth is via the causal pathway analysed here (that is, the decision between immediate birth or expectant management). Therefore, in our base case, we use ORs synthesised from the full intention‐to‐treat (ITT) populations of four RCTs for these outcomes.^12^, ^13^, ^14^, ^15^, ^16^, ^17^ We apply these to a pooled baseline probability for RDS and an estimate from NHS maternity statistics for caesarean birth.^12^, ^13^, ^18^ We test the impact of this assumption, using evidence from GBS‐positive subgroups only for all effectiveness parameters, with sensitivity analysis.

#### 2.1.2 Quality‐adjusted life years

The model estimates QALYs for both mothers and babies using UK‐based EQ‐5D data sources. For the mother, we apply utility decrements in the event of subsequent miscarriage or ectopic pregnancy. In the event of stillbirth, we apply a decrement reflecting the QALYs the baby would have accumulated over an average lifetime had they survived, equivalent to 25.06 discounted QALYs. We also apply a QALY decrement dependent on the duration of neonatal critical care to account for the extreme stress that parents experience (−0.0014 QALYs per day in a neonatal intensive care unit (NICU); see Appendix S3). The model does not account for the baby’s QALY loss as a result of the initial acute events, as the duration of these events is relatively short and there is no way of empirically quantifying health‐related quality of life in the affected babies. We calculate our utility multipliers for long‐term neurodevelopmental impairment using a recent UK study.^19^ Although this evidence comes from extremely preterm babies, in whom the rates and severity of neurodevelopmental disability are higher, there is no reason to believe that children classified as having mild, moderate or severe impairment will have meaningfully different prospects to those experiencing mild, moderate or severe impairment in the less premature population in which we are interested. Following standard advice, we assume the impact of multiple morbidities is multiplicative.^20^ To map morbidity following bronchopulmonary dysplasia onto health states for which we have utility values, we assume that ‘developmental delay’ equates to ‘mild neurodevelopmental impairment’, ‘neurological impairment’ equates to ‘moderate neurodevelopmental impairment’ and experiencing both equates to ‘severe neurodevelopmental impairment’. For the disutility of asthma, we use a study using English General Practice Patient Survey (GPSS) data.^21^ To account for changes in life expectancy for those with some level of disability we emulate the approach used in a recent cost‐effectiveness analysis,^22^ taking probabilities of death from 2016 to 2018 UK life tables and inflating them using hazard ratios from to estimate the additional risk of death as a result of neurodevelopmental impairment.^23^, ^24^

#### 2.1.3 Costs

We estimate antenatal care costs using reported resource use from the economic evaluation accompanying the PPROMT RCT and multiplying those by costs reported from NHS reference costs for 2016/2017 and subsequently inflating them to 2018/2019 prices.^5^, ^25^ The PPROMT data reflect all randomised participants, of whom only a minority had GBS; however, we take the view that GBS status alone is unlikely to have a substantial impact on antenatal resource use. We calculate delivery costs by estimating the proportion of caesarean and non‐caesarean births with each approach and applying unit costs for each from the NHS reference costs from 2016/2017. To estimate neonatal costs it would not be appropriate to use unadjusted resource‐use data from PPROMT, as the incidence of infections will be a key determinant of the costs and we need to reflect expected event rates in babies born to mothers testing positive for GBS. Therefore, we adopt a four‐stage approach: (i) we calculate the costs observed in the full PPROMT population in the same way as for antenatal costs;^5^ (ii) we estimate the additional costs associated with an average neonatal infection; (iii) we multiply this cost by the infection rate observed in the full trial population of PPROMT and deduct the result from the estimate calculated in step (i), to provide an estimate of the resource use and costs that would be expected if none of the babies experienced an infection; and (iv) we multiply our estimate of infection costs by the rates of infections we expect in each modelled arm of our GBS‐positive population and add those back on to our estimate of costs without infections, to provide estimates of the resource use and costs that correspond with the rate of infection in the model.

To estimate the costs for lifelong neurodevelopmental delay, we rely on publications from the EPICure longitudinal study of premature babies in the UK and Ireland.^19^, ^26^ As for quality of life, we assume that someone from our population of late‐preterm babies experiencing a specified level of impairment will incur similar costs to someone experiencing the same level of impairment secondary to extremely premature birth.

Finally, we use a weighted average of costs across different levels of control and frequency of exacerbations to estimate an annual cost for additional cases of asthma.^27^

### 2.2 Sensitivity analysis

To address parameter uncertainty in our base case, we performed one‐way and probabilistic sensitivity analyses. In one‐way sensitivity analysis, we vary each model input in turn within the range of its uncertainty (typically the lower and upper limit of its 95% confidence interval). In probabilistic sensitivity analysis, we assign distributions to each of the model inputs. The model draws estimates for each input simultaneously from their respective distributions and stores the results. We repeat this process for a set number of iterations (we selected 1000 iterations for our analysis). We plot the incremental costs and QALYs from each of these iterations on a cost–utility plane, indicating a ‘cloud’ of possible outcomes, given parameter uncertainty. We also present a cost‐effectiveness acceptability curve, showing the probability that each approach is optimal as we vary the value that we ascribe to 1 QALY.^28^ Table 1 details the values used for one‐way analyses and the distributions used in the probabilistic analysis.

## 3 RESULTS

### 3.1 Base case

**FIGURE 2:**
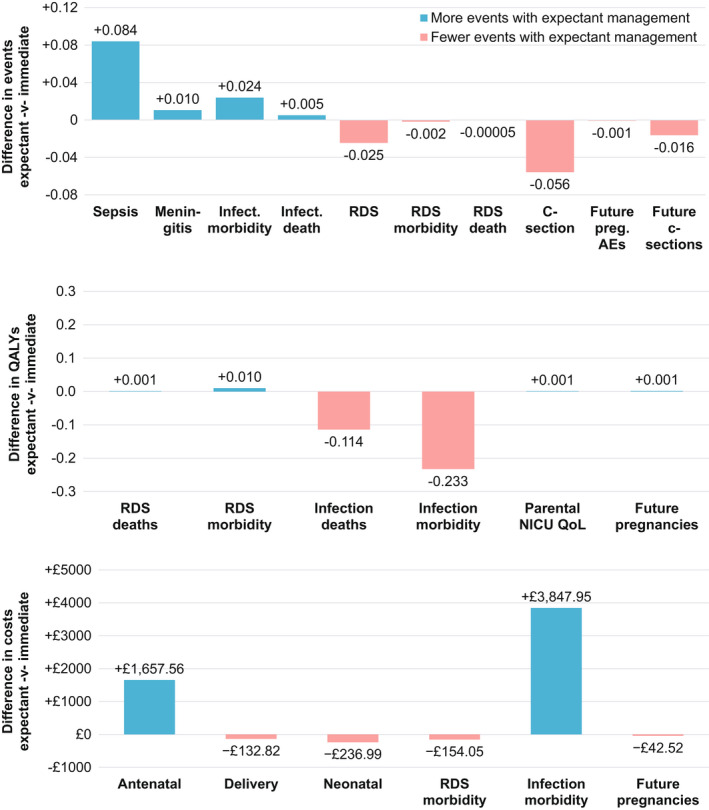
Differences in expected events, QALYs and costs per birth between the two approaches. AEs, adverse events; NICU, neonatal intensive care unit; QoL, quality of life; RDS, respiratory distress syndrome

Figure 2 shows differences in expected events, QALYs and costs per birth between the two approaches. Regardless of the approach, most babies experience no infection, no RDS and no long‐term morbidity. However, the proportion of deaths and morbidity associated with infection (favouring immediate birth) is clearly greater than the incidence of death and morbidity following RDS (favouring expectant management). Similarly, although expectant management is associated with more QALYs and lower costs when it comes to peri‐ and postnatal care, the sequelae of RDS and consequences for future pregnancies, these impacts are dwarfed by the QALY losses and increased costs that occur because of the additional neonatal infections associated with immediate birth.

Bringing these together, we estimate that immediate birth results in average costs of £14,372 and average QALYs of 24.705, over the baby’s lifetime, whereas expectant management costs £19,311 and generates 24.371 QALYs, on average. As such, immediate birth is the dominant option, resulting in reduced costs and more QALYs (incremental costs of –£4939 and incremental QALYs of 0.333, equivalent to an extra third of a year in perfect health per birth).

In one‐way sensitivity analysis (see Appendix S5) there is only one model input that, when varied within its plausible bounds, could make expectant management the preferred approach: the OR estimating the relative effect of the approach on the incidence of infections. At the lower bound of the 95% confidence interval, the data for this parameter are consistent with expectant management resulting in fewer infections (that is, the lower end of the interval is <1). If this were the true value of the parameter, the model would favour expectant management, as all outcomes (infections, RDS and delivery) would favour that approach over immediate birth. On detailed inspection (see Appendix S5), we found that the odds of infection only have to be ≥1.5% higher with expectant management for the benefit of avoiding infection to outweigh the other disadvantages of immediate birth. Restricting all effectiveness data to only mothers who test positive for GBS did not materially affect the results.

**FIGURE 3:**
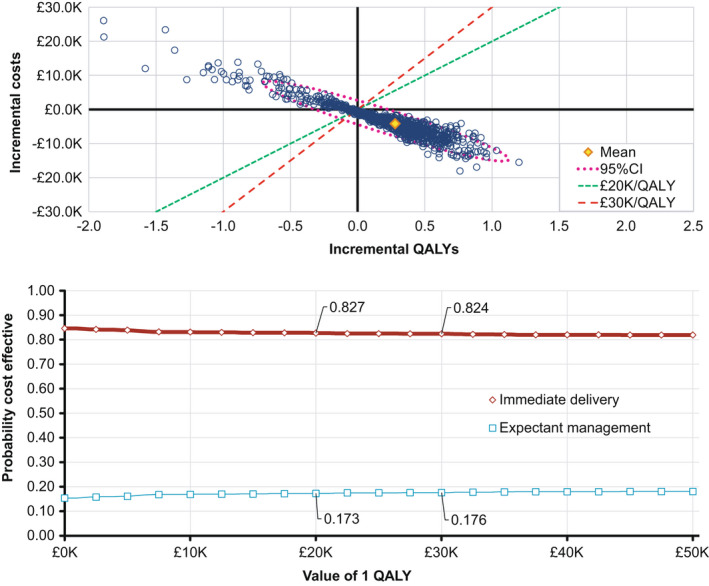
Probabilistic sensitivity analysis plots. Upper panel, cost–utility scatter plot; lower panel, cost‐effectiveness acceptability curve

Probabilistic sensitivity analysis (Figure 3) shows substantial correlation between costs and QALYs. This is, once more, a result of the predominance of the OR for infection in determining the model outputs: when a high OR is sampled, immediate birth is associated with both lower costs and higher QALYs; when a low OR is sampled, that relationship is reversed. The cost‐effectiveness acceptability curve (CEAC) is characteristic of an economic analysis with these conditions. The value that we place on QALYs has almost no effect on which option is considered optimal; this is because, in any given simulation, the model predicts either that immediate birth dominates expectant management or that expectant management dominates immediate birth. As the probability mass in the distribution for the infection OR strongly favours immediate birth, over 80% of simulations suggest that this is the preferred option.

## 4 DISCUSSION

### 4.1 Main findings

This economic analysis from a UK NHS and PSS perspective shows that, for women who test positive for GBS with PPROM at 34^+0^–36^+6^ weeks of gestation, immediate birth dominates expectant management: it is both cheaper and provides more QALYs. These results arise because our model predicts that the lifetime discounted costs and consequences associated with neonatal GBS infection far outweigh those that can be expected from the complications of late‐preterm birth. Our calculations quantify the potentially ruinous consequences of neonatal GBS infection to babies, families, and the health and care system: the model estimates that an average infection is associated with discounted lifetime costs of approximately £40,000 and discounted lifetime effects of about 3.8 QALYs lost (undiscounted figures are approx. £130,000 and 11.0 QALYs lost). This is why, even though the expected rates of infection are relatively low with either approach, immediate delivery is still associated with a substantial incremental QALY gain of 0.333 and cost savings of £4939, compared with expectant management for each case of PPROM. Sensitivity analysis shows that the OR estimating the relative likelihood of infection between the two approaches is the only meaningful contributor to decision uncertainty. At a 95% confidence level, the RCT data do not exclude either approach having a higher rate of infections; if there really were fewer infections with expectant management, it would become the preferred option.

### 4.2 Strengths and limitations

This is the first economic analysis of this decision problem (focusing on women with PPROM who test positive for GBS) and the first of any type of late‐preterm PPROM to estimate QALYs, accounting for lifelong morbidity and mortality. Its development was informed by a multidisciplinary committee of clinical and patient experts who advised on structure, assumptions and potential data sources, and provided validation of the model outputs.

An ideal model of this problem would use evidence directly reporting lifelong effects of the two approaches. However, no such data exist. Our challenge was to move from the short‐term outcomes reported in RCTs to costs and QALYs over a lifetime. Estimating the impact of neonatal infections using observational evidence describing the mortality and morbidity with which events are associated is an established approach.^22^, ^29^, ^30^, ^31^ Our methods for estimating the long‐term consequences of late‐preterm birth are more innovative, using the incidence of RDS as a proxy measure, to tie long‐term outcomes to an outcome observed in the RCTs. To do this, we use evidence on chronic lung disease and its consequences. However, this comes from cohorts that, although they do not exclude late‐preterm babies, predominantly represent more premature babies. We recognise that, in the UK, bronchopulmonary dysplasia is seldom used as a diagnosis in late‐preterm babies (although, clearly, such babies sometimes require prolonged oxygen support, which is the primary diagnostic criterion in most definitions of bronchopulmonary dysplasia). However, we adjusted for gestational age when assessing this outcome, and we note that a large RCT in babies born at 34^+0^–36^+6^ weeks of gestation found a bronchopulmonary dysplasia incidence rate of 0.6% in its control arm;^32^ this is identical to the rate we predict for the immediate birth arm of our model. Therefore, although it relies on some evidence from outside our late‐preterm population, we are reasonably confident that our approach appropriately enables us to take advantage of a short‐term outcome that is reported in relevant RCTs to estimate lifelong impacts.

Compared with many analyses of its type, our decision model has relatively few inputs that rely on expert consensus. However, we were unable to locate suitable estimates for the quality‐of‐life impacts of miscarriage, ectopic pregnancy and stillbirth in future pregnancies. To ensure our results are robust to the assumptions that we adopted, we tested the sensitivity of our model to extreme values and found that these have a negligible impact on the outputs (see Appendix S5). Nevertheless, these are clearly major events that feature in a wide variety of obstetric decision problems, so research on the utility values with which they are associated is sure to enhance future analyses like ours.

This analysis takes a UK NHS perspective, and the balance of benefits, harms and costs may vary among countries. Nevertheless, our sensitivity analyses suggest that in any setting in which immediate birth can be assumed to prevent infections it is likely to represent an effective use of resources, compared with expectant management. We have shown (Appendix S5) that our results are relatively robust to the most likely differences between jurisdictions: a higher or lower underlying rate of infections; costs of immediate and/or long‐term treatment; and societal preferences for different outcomes. The only parameter that drives results is the extent to which immediate birth reduces infections, and we think this is the input that is most likely to generalise across settings. However, we cannot say with certainty that our results are generalisable outside the UK without redoing our analysis with a comprehensive set of country‐specific input parameters.

### 4.3 Interpretation

We suggest that, regardless of any statistical uncertainty about which approach results in fewer infections, it is hard to specify plausible mechanisms by which immediate birth might increase the risk of infection to a greater degree than expectant management. If a woman with ruptured membranes is colonised with GBS the fetus remains in an environment in which both the potential pathogen and a portal for transmission are present, but this is less likely with immediate birth. It follows that infections must, to some degree, be more common with expectant management compared with immediate birth. Although we are uncertain about the magnitude of this effect, the model shows that the odds of GBS infection only need be 1.5% higher with expectant management for immediate birth to be the preferred approach.

The NICE methods dictate that decision makers should consider QALYs as ‘of equal value regardless of other characteristics of the individuals, such as … age’.^9^ However, some research suggests that society may value children’s QALYs more highly than those of adults, although this has not been a universal or straightforward finding.^33^ Applying different weights to different people’s outcomes might be challenging in an analysis like this, where net results comprise a mixture of maternal QALYs, neonatal QALYs and the QALYs that people who are babies at the time of the decision can expect to accrue as children and adults. However, in practice, this debate has almost no impact on the decision problem at hand: as illustrated in Figure 3 and explained above, our analysis is essentially invariant to the value that we place on QALYs.

Our analysis only addresses a population in which the maternal GBS status is known. Although the routine antenatal screening of *all* pregnant women for GBS colonisation is not currently recommended in the UK,^34^ ad hoc testing is variably undertaken in the NHS. Although the NICE guideline did not look at the cost‐effectiveness of testing, it might be inferred that because (i) our results suggest that immediate birth is associated with better outcomes and (ii) appropriately obtained and analysed GBS tests are relatively inexpensive and accurate, performing such tests is likely to be an effective use of NHS resources. We would expect the benefits shown in this analysis to be enough to justify this, even without accounting for the possible benefit of intrapartum antibiotics. Results from continuing RCTs will be important to confirm or contradict this hypothesis.^35^ Pending such data, clinicians will need to take a view about whether the observation of increased risk in the presence of known GBS in women with PPROM at 34^+0^–36^+6^ weeks of gestation constitutes a ‘material fact’ in a post‐Montgomery era. If so, testing and an opportunity to undertake it with a full discussion of the implications of a positive or negative result should be a decision for the woman rather than the clinician.

## 5 CONCLUSION

Our analysis suggests that, for babies at 34^+0^–36^+6^ weeks of gestation, the lifetime costs and harms of neonatal infection far outweigh those of preterm birth. Therefore, for women with PPROM who test positive for GBS, immediate birth, which minimises the risk of neonatal infection, generates better expected health outcomes and lower costs than expectant management.

## DISCLOSURE OF INTERESTS

JD, PB and GR have no conflicts of interest to declare. FC works at Novartis. The views expressed in this study are those of FC and not necessarily those of Novartis. SC‐D works at BresMed. The views expressed in this study are those of SC‐D and not necessarily those of BresMed. JP is the Chief Executive employed by Group B Strep Support, the Vice‐Chair of the RCOG Women’s Network and is a co‐applicant on GBS3 (https://www.journalslibrary.nihr.ac.uk/programmes/hta/178606/#/) and iGBS3 (https://clinicaltrials.gov/ct2/show/NCT04735419). AS is a member of both the RCOG scientific advisory committee and the Royal College of Paediatrics and Child Health (RCPCH) quality improvement committee, and is the clinical lead at both the Kent and Medway Local Maternity system and the NHS South East Coast Neonatal Network. Completed disclosure of interests form available to view online as supporting information.

## AUTHOR CONTRIBUTIONS

GR, JP, PB and AS designed the analysis, with the help of the other NICE committee members acknowledged above. GR, FC, SC‐D and JD found parameters. GR and JD and analysed the data. JD, GR, JP, PB and AS provided the interpretation of results. JD wrote the first draft of the article. All authors critically revised the article and approved the final version for publication.

## ETHICAL APPROVAL

As no human participants were involved in this theoretical analysis, no approval from an institutional review board was sought.

## Supporting information


**Appendix** S1

Data S1

Data S2

Data S3

Data S4

Data S5

Data S6

Data S7

## Data Availability

Data sharing is not applicable to this article as no new data were created or analysed in this study.
