# Supplementary material for: Immediate birth for women between 34 and 37 weeks of gestation with prolonged preterm prelabour rupture of membranes and detection of vaginal or urine group B streptococcus: an economic evaluation
Source: BJOG. 2022 Mar 8;129(10):1779–89. doi: 10.1111/1471-0528.17119 (PMC9543209; doi:10.1111/1471-0528.17119)
Supplement: Supplementary file 1 — Appendix S1 [file BJO-129-1779-s008.docx]

**Immediate birth for women between 34–37 weeks’ gestation with preterm prelabour prolonged rupture of membranes and vaginal or urine GBS detection: an economic evaluation**

Jeremy Dietz, Jane Plumb, Philip Banfield, Aung Soe, Fadi Chehadah, Stacey Chang-Douglass, Gabriel Rogers

**ELECTRONIC SUPPLEMENTARY MATERIAL**

**Contents**

[Appendix S1: Derivation of model parameters – natural history 2](#_Toc89417036)

[S1.1 Baseline probability of infection 2](#_Toc89417037)

[S1.2 Conditional probability of meningitis given infection 2](#_Toc89417038)

[S1.3 Sequelae of infection 3](#_Toc89417039)

[S1.4 Baseline probability of RDS 5](#_Toc89417040)

[S1.5 Conditional probability of BPD given RDS 5](#_Toc89417041)

[S1.6 Sequelae of BPD 6](#_Toc89417042)

[S1.7 Baseline probability of caesarean birth 7](#_Toc89417043)

[S1.8 Consequences of caesarean birth 7](#_Toc89417044)

[Appendix S2: Derivation of model parameters – effects of interventions 10](#_Toc89417045)

[S2.1 Infection 10](#_Toc89417046)

[S2.2 Respiratory distress syndrome 10](#_Toc89417047)

[S2.3 Caesarean birth 11](#_Toc89417048)

[Appendix S3: Derivation of model parameters – quality of life 12](#_Toc89417049)

[S3.1 Impact of neonatal critical care 12](#_Toc89417050)

[S3.2 Long-term consequences of infection / sepsis 13](#_Toc89417051)

[S3.3 Long-term consequences of bronchopulmonary dyplasia 14](#_Toc89417052)

[S3.4 Consequences of caesarean birth for future pregnancies 15](#_Toc89417053)

[Appendix S4: Derivation of model parameters – costs 16](#_Toc89417054)

[S4.1 Antenatal care 16](#_Toc89417055)

[S4.2 Delivery costs 17](#_Toc89417056)

[S4.3 Neonatal costs 18](#_Toc89417057)

[S4.4 Total perinatal costs 20](#_Toc89417058)

[S4.5 Long-term morbidity – cost per year 21](#_Toc89417059)

[S4.6 Consequences of caesarean birth for future pregnancies 23](#_Toc89417060)

[Appendix S5: Supplementary results 26](#_Toc89417061)

[S5.1 One-way sensitivity analysis 26](#_Toc89417062)

[S5.2 Threshold analysis 28](#_Toc89417063)

[S5.3 Additional probabilistic sensitivity analyses 28](#_Toc89417064)

[References 33](#_Toc89417065)

# Derivation of model parameters – natural history

## Baseline probability of infection

As shown in Table S1.01, the model assumes baseline infection risk (for expectant management) of 15% (7/46), taken from risk of early-onset infection in mothers colonised with GBS in Tajik et al. (1). We preferred this source as the population had prolonged (>24-hour) rupture of membranes, in line with our decision problem. This was not an eligibility criterion in a larger RCT (2), which included all cases with clinically suspected rupture of membranes. While we assume this distinction is unlikely to have any meaningful influence on the **relative** effects of the 2 approaches (therefore, we are happy to pool data from both for that purpose; see S2.1), the same cannot be said of the **absolute** probability of infection. Therefore, we selected the higher risk of infection observed in Tajik et al. (1) as the more appropriate value for our population. We explore the impact of abandoning this distinction, by using a pooled estimate from GBS+ subgroups of both RCTs in a scenario analysis.

Table S1.01: Infection risk

|  | Risk | Source |
| --- | --- | --- |
| Base case | | |
| GBS+, prolonged PPROM | 15.2% (7/46) | (1) |
| Alternative value (scenario analysis) | | |
| GBS+ trial-arms pooled^a^ | 7.9% (5.3% to 17.2%) | (1) & (2) |
| 1. fixed-effect meta-analysis on log-odds scale | | |

## Conditional probability of meningitis given infection

As done in prior analyses of neonatal infection (3,4) the model subdivides infections into meningitis and sepsis. In order to do this, the model requires an estimate of the probability that any given infection will be meningitis, with sepsis assumed to represent the remainder of cases (this is consistent with the definitions used in the RCTs, which required clinical symptoms to be present to classify a case as an infection).

Table S1.02 summarises the different potential sources for conditional probability of meningitis, given infection. For our base case, we assume a 11% probability, which we took from a surveillance cohort in the UK and Ireland (5). Because other values we identified for the same parameter were very similar, we did not explore them as alternative model inputs.

Table S1.02: Risk of meningitis

|  | Risk of meningitis | Source |
| --- | --- | --- |
| Base case | | |
| Given early onset GBS | 0.110 (57/517) | (5) |
| Alternative values (not used) | | |
| Given early onset GBS | 0.118 (12/102) | (6) |
| Given early onset GBS preterm | 0.101 (95% CI 0.056 to 0.156) | (3) |
| Given early onset GBS term | 0.119 (95% CI 0.081 to 0.164) | (3) |

## Sequelae of infection

### Death from neonatal meningitis

To predict the likelihood of death in neonates who contract meningitis, the model uses data from a surveillance cohort in the UK and Ireland (7). This evidence shows that risk of death is strongly associated with gestational age. Therefore, we calculate separate case-fatality rates for our 2 cohorts, using the proportion of babies born at less than 37 weeks’ gestational age from Morris et al. (2). This leads to a somewhat higher risk of death in the immediate birth arm, in which almost all neonates were born prematurely, than in the expectant management arm, where some babies reached term.

Previous analyses (8) have used data from Colbourn et al.’s multiparameter evidence synthesis (3) to estimate this parameter. We explore the use of these alternative values in sensitivity analysis. The study estimates case-fatality probabilities for both term and preterm babies; however, in this case, preterm cases include very premature babies that are outside our decision-space. Therefore, we felt that it would be most appropriate to use estimates for term babies alone.

Table S1.03: Death from neonatal meningitis

|  | Risk of death | Source |
| --- | --- | --- |
| Base case | | |
| 32–36 weeks’ gestation | 9.3% (4/43) | (7) Tab 3 |
| 37+ weeks’ gestation | 4.3% (10/235) | (7) Tab 3 |
| Weighted average for each approach: |  |  |
| Immediate (96.9% <37wk) | 9.1% |  |
| Expectant (79.4% <37wk) | 8.3% |  |
| Alternative value (scenario analysis) | | |
| Early onset GBS meningitis term | 0.124 (95%CI: 0.027 to 0.277) | (3) Tab 26 |
| Late onset GBS meningitis term | 0.111 (95%CI: 0.037 to 0.216) | (3) Tab 26 |

### Death from neonatal sepsis

We used a similar approach to estimate the probability of death from sepsis without meningitis. Data from the same surveillance unit (5) provide outcome data for 856 cases of invasive GBS that was predominantly classified as sepsis. As for meningitis, risk of death is strongly associated with gestational age, and we account for this in the same way, by weighting gestation-specific risks by probability of preterm birth in each arm (see Table S1.04).

However, this study also includes a small proportion of neonates with GBS-related meningitis (57 of 517 cases with 3 of 27 deaths), which we would ideally like to exclude from this model parameter, and only presents gestation-stratified case-fatality results in this mixed cohort. We are able to exclude the cases from the overall death-rate, though we are not able to account for gestational age if we do so, so we include a single fatality-rate for both arms as a sensitivity analysis. In addition, we explore the data from Colbourn et al. (3), as before.

Table S1.04: Death from neonatal sepsis

|  | Risk of death | Source |
| --- | --- | --- |
| Base case | | |
| 34–36 weeks’ gestation | 6.1% (3/49) | (5) |
| 37+ weeks’ gestation | 2.7% (9/330) | (5) |
| Weighted average for each approach: |  |  |
| Immediate (96.9% <37wk) | 6.0% |  |
| Expectant (79.4% <37wk) | 5.4% |  |
| Alternative value (scenario analysis) | | |
| All gestational ages, no meningitis | 5.2% (24/460) | (5) |
| Early onset GBS sepsis term | 0.053 (95%CI: 0.025 to 0.088) | (3) Tab 26 |
| Late onset GBS sepsis term | 0.061 (95%CI: 0.012 to 0.141) | (3) Tab 26 |

### Risk of disability due to infection

The model assumes that infections may lead to long-term disability. We took the risks of disability due to infection from the same NIHR-funded evidence synthesis that was used to estimate sequelae in NICE CG149 (3,4), as summarised in Table S1.05. The analysis applies separate disability risks for meningitis and sepsis without meningitis.

Table S1.05: Risk of disability due to infection - from Colbourn et al. (3)

|  | Risk of disability (95% CI) | |
| --- | --- | --- |
|  | Meningitis | Sepsis without meningitis |
| No disability | 0.614 (0.535 to 0.692) | 0.746 (0.641 to 0.838) |
| Mild disability | 0.196 (0.136 to 0.264) | 0.045 (0.011 to 0.100) |
| Moderate disability | 0.129 (0.081 to 0.187) | 0.139 (0.072 to 0.222) |
| Severe disability | 0.061 (0.029 to 0.104) | 0.070 (0.023 to 0.138) |

### Expected lifespan of neonatal survivors

We also need an estimate of expected lifespan to estimate the costs and effects for neonates sustaining lifelong morbidity. For this, we emulate the approach used in a recent cost-effectiveness analysis (9).  This approach takes the probability of death from 2016–18 UK life tables (10) and inflates it using hazard ratios from Reid et al. (11) to estimate the additional risk of death due to NDI. Table S1.06 shows the resulting estimates.

Table S1.06: Expected lifespan of neonatal survivors

| Severity of impairment | Hazard ratio (95%CI) (11) | Equivalent life expectancy at birth using 2016–18 UK lifetables (10) | | |
| --- | --- | --- | --- | --- |
|  |  | Undiscounted | Discounted | |
|  |  |  | 3.5% / year | 1.5% / year |
| Motor impairment | | | | |
| None |  | 81.04 | 27.40 | 46.89 |
| Mild | 1.00 | 81.04 | 27.40 | 46.89 |
| Moderate | 1.51 (0.71 to 3.24) | 76.82 | 27.02 | 45.48 |
| Severe | 6.21 (3.28 to 11.77) | 60.59 | 24.87 | 39.13 |
| Intellectual impairment | | | | |
| None | 1.00 | 81.04 | 27.40 | 46.89 |
| Mild–moderate | 1.11 (0.62 to 1.97) | 79.98 | 27.31 | 46.55 |
| Severe–profound | 3.01 (1.74 to 5.22) | 69.29 | 26.17 | 42.73 |

## Baseline probability of RDS

The model assumes baseline RDS risk (for immediate birth) of 8.0%, taken from a pooled analysis of the immediate birth arms from the RCTs (Table S1.07). We assume that there is no reason to suspect that the mother’s GBS status would have any meaningful effect on the probability that their baby will experience RDS. Therefore, we pool data from the full sample of each RCT. Data from Tajik et al. (1) can be stratified according to maternal GBS status, and confirm our assumption that there is unlikely to be a meaningful difference in RDS rates according to this factor.

Table S1.07: Probability of RDS

|  | Risk |
| --- | --- |
| Morris et al. (2) | 8.3% (76/919) |
| Tajik et al. (1) (GBS+ only) | 8.8% (5/57) |
| Tajik et al. (1) (GBS-) | 6.9% (21/306) |
| Trial-arms pooled^a^ | 8.0% (95%CI: 6.6 to 9.6%) |
| 1. fixed-effect meta-analysis performed on log-odds scale before transforming back to natural probabilities | |

## Conditional probability of BPD given RDS

The model assumes that a proportion of babies with RDS will develop BPD, which may, in turn, lead to mortality and long-term morbidity. Consequently, we require an estimate of the conditional probability of BPD given RDS. We were unable to find any published research directly addressing this question in the population in which we are interested (that is, relatively late-preterm babies). Some literature looks at the incidence of BPD among all preterm babies with RDS; however, because gestational age is a critical determinant of this outcome, we had to adjust our estimates to be representative of the population of interest. Fortunately, it is clear that the probability of BPD given RDS follows an approximately logistic distribution with respect to birthweight – that is, the log-odds of BPD have a linear relationship with birthweight; Horbar et al. (12) – and we were content to assume that a similar relationship holds for gestational age.

Therefore, we were able to base our calculations on a prediction model for BPD by Zysman‑Colman et al. (13). We take 3 datapoints from this study: the prevalence of BPD among all premature babies with RDS is 36% (806 out of 2,233 cases), the mean gestational age is 31.2 weeks and the odds ratio for BPD per additional week of gestation is 0.62 (95%CI: 0.60 to 0.64). Using these data (and the assumption of a logistic relationship between gestational age and probability of BPD), we can estimate *o*_(BPD|RDS,_ *_x_*_)_ – the odds that a child born with RDS at gestational age *x* will develop BPD:

| $\text{o}_{\text{(BPD\vert RDS, }\text{x}\text{)}}\text{ }\text{=}\text{ }\frac{\text{0.36}}{\text{1-0.36}}\text{0.62}^{\text{(}\text{x}\text{-31.2)}}$ | **(1)** |
| --- | --- |

And then a standard odds-to-probability transformation gives us *p*_(BPD|RDS,_ *_x_*_)_ – the probability a child with RDS born at gestational age *x* will develop BPD:

| $\text{p}_{\text{(BPD\vert RDS, }\text{x}\text{)}}\text{ }\text{=}\text{ }\frac{\text{o}_{\text{(BPD\vert RDS, }\text{x}\text{)}}}{\text{1+}\text{o}_{\text{(BPD\vert RDS, }\text{x}\text{)}}}$ | **(2)** |
| --- | --- |

By design, gestational age will be different in an immediate birth strategy than with expectant management. Therefore, the probability that neonates experiencing RDS will go on to develop BPD will also vary between the 2 approaches. To capture this, we used the mean gestational ages from Morris et al. (2): mothers randomised to immediate birth gave birth at an average of 35.1 week’s gestation whereas, for the expectant management strategy, the equivalent value was 35.6 weeks. Plugging these numbers into equations (1) and (2) gives final estimates for the model: *p*_(BPD|RDS, 35.1)_ = 0.0798 for immediate birth and *p*_(BPD|RDS, 35.6)_ =0.0635 for expectant management.

## Sequelae of BPD

### Death related to BPD

The model also captures the additional mortality associated with BPD. As described above, we preferred to assume that all cases of BPD are mild in our base-case model. Landry et al. (14) reported a mortality risk of 2% (1/60) among mild BPD patients. We test this in sensitivity analysis using data on all severities of BPD from the same study (noting that this includes a large proportion of infants who were born much more prematurely than our cohort): 16.5% (53/322).

### Consequences of bronchopulmonary dysplasia (BPD)

The model assumes that the proportion of neonates who have RDS and go on to develop BPD are at risk of lifelong sequelae. The best source of evidence we identified for this was a Canadian case series reported by Landry et al. (14), reviewing children with BPD after 2–5 years’ follow-up. In their study, the 3 most prevalent complications are developmental delay, neurological impairment and wheezing episodes/asthma. Thus, we model these three complications as the possible morbidities neonates with BPD may develop.

Landry et al. (14) stratify risk of long-term complications according to BPD severity. However, the cohort is, on average, more premature than our model population (28 weeks versus 34+ weeks gestational age). Therefore, in our base case, we assume that all cases of BPD are mild; we explore the impact of this assumption by using the risks across all severities of BPD in a scenario analysis. Table S1.08 summarises the inputs.

Table S1.08: Long-term complications related to BPD - from Landry et al. (14)

|  | Mild BPD (base case) | All BPD (scenario analysis) |
| --- | --- | --- |
| Developmental delay | 34.3% (12/35) | 52.4% (87/166) |
| Neurological impairment | 14.3% (5/35) | 20.4% (33/162) |
| Wheezing episodes/ asthma | 35.9% (14/39) | 34.3% (35/102) |
| NB denominators not specified in original article, but possible to infer from published event-counts and percentages | | |

## Baseline probability of caesarean birth

The model assumes a baseline caesarean birth probability of 30.1% for expectant management. This figure comes from NHS maternity statistics 2018–19 (15), which reports 179,475 caesareans among 596,101 total births for which mode of delivery is recorded. The included RCTs (1,2) from the systematic review undertaken as part of NICE’s guideline on Neonatal infection: antibiotics for prevention and treatment ([NG195](https://www.nice.org.uk/guidance/ng195)) (16) report a lower probability of caesarean births in their expectant management arms. However, these trials report predominantly non-UK practice (Tajik et al. (1) is Dutch; Morris et al. (2) is international, with mostly Australian participants), and we assume that rates of caesarean birth are highly dependent on prevailing practice in the country in question. Therefore, we use the NHS maternity statistics estimate – which has the advantage of being UK-specific but has the disadvantage of not being drawn from the subpopulation in which we are interested – for our base case, and explore the impact of the RCT-derived estimates in sensitivity analysis (see Table S1.09).

Table S1.09: Probability of caesarean birth

|  | Risk |
| --- | --- |
| Base case | |
| NHS maternity statistics (2018–19) (15) | 30.1% (179,475/596,101) |
| Alternative value (scenario analysis) | |
| Morris et al. (2) | 18.5% (169/912) |
| Tajik et al. (1) (GBS+ only) | 15.2% (7/46) |
| Tajik et al. (1) (GBS-) | 15.0% (47/313) |
| Trial-arms pooled^a^ | 17.6% |
| 1. fixed-effect meta-analysis on log-odds scale | |

## Consequences of caesarean birth

### Probability of future pregnancies

Using ONS childbearing data (17), we calculate that 55% of live births will have at least 1 subsequent live birth. The mean number of expected future live births, among women who have at least 1 more child, is 1.46. 14.3% of pregnancies will not result in a live birth post-caesarean (Table S1.12); therefore, 1.704 pregnancies would occur to produce 1.46 live births.

### Interval until future pregnancy

In order to discount the costs of future pregnancies appropriately we also need to understand the expected length of time between pregnancies. ONS birth interval figures shown that the median birth interval is 35 months (18).

Table S1.10: Expected future births

| Expected future births | Proportion of women | Median birth interval | Proportion of future births |
| --- | --- | --- | --- |
| 1 | 100% | 35 | 68% |
| 2 | 36% | 70 | 25% |
| 3 | 10% | 105 | 7% |

By combining this with the number of future expected births (if>0), we can estimate the mean birth interval until a future birth as:

35 × 0.68 + 70 × 0.25 + 105 × 0.07 = 48.5 months

This is equal to 4.04 years.

### Probability of future caesarean

The clearest consequence of a caesarean birth is that it substantially raises the chances that any future babies the mother has will also be delivered by caesarean. Data from the NHS Maternity Audit (19) show that the rate of vaginal birth after caesarean (VBAC) is 24.9%; we use the complement of this value directly to estimate the probability of caesarean in all future births for women whose current baby is born by caesarean. However, to quantify how much a caesarean in the current birth raises this probability, we also need to know what the probability of caesarean would have been if the current baby had not been born by caesarean. We approximate this figure using data from NHS maternity statistics (15). We multiply the proportion of women who did not have a VBAC by the proportion of women who had a caesarean for their first birth: 0.749 × 0.306 = 22.9%. We then assume that the remaining caesareans came from mothers who did not have a caesarean for their first child; see Table S1.11.

Table S1.11: Mode of delivery for subsequent pregnancies

| Type | Value | Source / derivation |
| --- | --- | --- |
| VBAC (a) | 25.1% (12,449/49,542) | (19) |
| Primiparous caesareans (b) | 30.6% (46,839/153,279) | (15) |
| Multiparous caesareans (c) | 30.3% (39,240/129,364) | (15) |
| As proportion of multiparous births |  |  |
| Caesarean after caesarean (d) | 22.9% | b × (1−a) |
| Caesarean after non-caesarean (e) | 7.5% | c−d |
| Non-caesarean after caesarean | 7.7% | b × a |
| Non-caesarean after non-caesarean | 62.0% | (1−b)−e |
| Probabilities |  |  |
| Caesarean given prior caesarean | 0.749 | 1−a |
| Caesarean given no prior caesarean | 0.107 | (c−d) / (1−b) |

### Probability of adverse outcomes

The model also uses evidence that women who have had a caesarean birth are at higher risk of ectopic pregnancy, miscarriage or stillbirth in future pregnancies, based on a published meta-analysis (20).

The model applies these relative effects to estimates of absolute risk of each event drawn from the literature:

- 1.1% for ectopic pregnancy; following NICE NG126 (21), we draw this estimate from a 3-year review of adverse pregnancy events in Britain and Ireland (22).
- 12.8% for miscarriage, based on a large, recent cohort study from Norway (23).
- 4.1 stillbirths per 1,000 total births in England, based on ONS 2017 data (24).

However, each of these absolute risks represents a mixture of women who have not undergone a previous caesarean birth and those who have. We need to adjust for this to arrive at a best estimate of event-rates with and without the exposure. We do this using 3 pieces of information: the observed probability in all women (which we convert to odds), the odds ratio for exposed -v- unexposed, and an estimate of the proportion of women who have the exposure. From the NHS maternity statistics 2018–19 (15), we estimate that approximately one-fifth of pregnant women have a history of caesarean birth (82,949 ÷ 426,698 = 19.4%; 82,949 = [421,552 births − 153,279 to exclude primiparous] × 0.306 [b in Table S1.11]).

Using these 3 values, we note that the observed odds of experiencing the event (o_all_) are a combination of the odds with the exposure (o_CS_) and odds without the exposure (o_noCS_) weighted according to the probability of exposure (p_CS_):

| $o_{all}=o_{CS}p_{CS}+o_{noCS}\left( 1-p_{CS} \right)$ | **(3)** |
| --- | --- |

And the relation between the exposed and unexposed odds is defined by our odds ratio (OR_CS‑v‑noCS_):

| $o_{CS}=o_{noCS}{OR}_{CSvnoCS}$ | **(4)** |
| --- | --- |

These 2 expressions may be treated as simultaneous equations and rearranged as:

| $o_{noCS}=\frac{o_{all}}{\left( 1-p_{CS} \right)+p_{CS}{OR}_{CSvnoCS}}$ | **(5)** |
| --- | --- |

Once we have a result for the unexposed, we plug it into equation (4) to estimate odds in the exposed. Finally, we convert the resulting odds to probabilities. The results of these calculations are shown in Table S1.12.

Table S1.12: Future pregnancy events

| Event | Baseline probability | Source | Odds ratio prev. caesarean -v- none (95%CI) | Source | Probability according to prev. caesarean | |
| --- | --- | --- | --- | --- | --- | --- |
|  |  |  |  |  | No | Yes |
| Miscarriage | 12.8% (53,906 / 421,201) | (23) | 1.21 (1.04 to 1.40) | (20) | 12.4% | 14.6% |
| Ectopic | 1.1% (32,100 / 2,891,892) | (22) | 1.17 (1.03 to 1.32) | (20) | 1.07% | 1.26% |
| Stillbirth | 0.41% (2,689 / 659,765) | (24) | 1.27 (1.15 to 1.40) | (20) | 0.39% | 0.49% |

# Derivation of model parameters – effects of interventions

## Infection

For risk of infection, we decided that our estimate of relative effect should come from the population directly reflecting our decision problem: that is, women with preterm, prelabour rupture of membranes with GBS detection. Therefore, we took this value from the systematic review undertaken as part of NICE’s guideline on Neonatal infection: antibiotics for prevention and treatment ([NG195](https://www.nice.org.uk/guidance/ng195)) (16) (Figure S2.01), which showed that expectant management is associated with a relative risk of infection of 2.73 (95%CI: 0.34 to 22.18) compared with immediate birth. The equivalent odds ratio is 2.93 (95%CI: 0.33 to 26.19). Because we decided that it would not be appropriate to use infection rates from women without GBS detection, we do not use those data even for sensitivity analyses.

Figure S2.01: Treatment effects (expectant management -v- immediate birth): infection

| 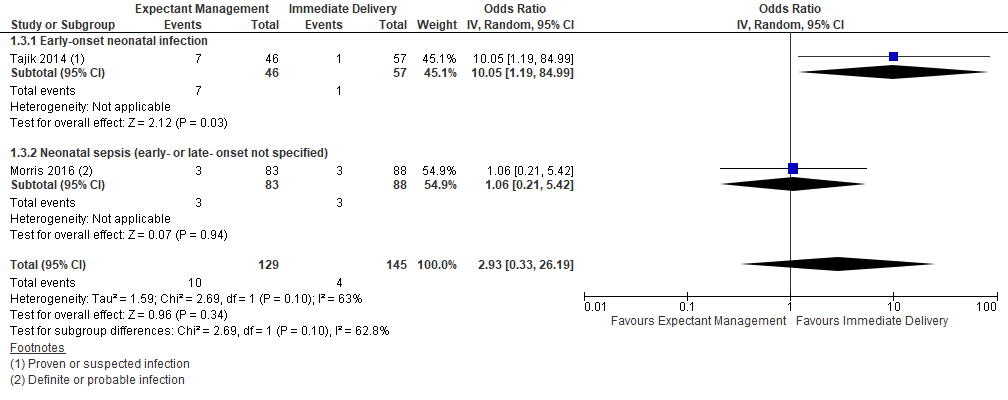 |
| --- |

Table S2.01: Treatment effects (expectant management -v- immediate birth): infection

|  | GBS status | N RCTs | Odds ratio (95%CI) | *I* ^2^ | Model |
| --- | --- | --- | --- | --- | --- |
| **Included RCTs (base case)** | **GBS+ only** | **2** | **2.93 (0.33 to 26.19)** | **63%** | **RE** |
| Included RCTs | All | not appropriate for this outcome | | | |
| Cochrane review | All |  |  |  |  |

## Respiratory distress syndrome

For risk of RDS, we decided again to pool evidence from GBS-positive women with data from groups in which GBS status was negative or unknown. Results are shown in Figure S2.02. Here, there is a good degree of agreement between the datapoints, all of which show that immediate birth is associated with higher rates of RDS, regardless of mothers’ GBS status.

Figure S2.02: Treatment effects (expectant management -v- immediate birth): respiratory distress syndrome

| 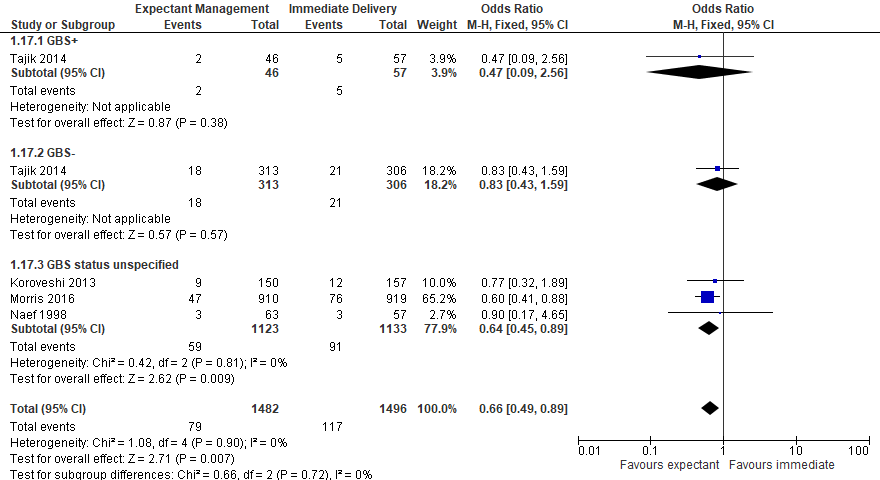 |
| --- |
| Note that the pooled total in this graph is very slightly different from the estimate from the Cochrane review we use in our model because it shows GBS-stratified results from Tajik et al. (1), to explore evidence for GBS-related heterogeneity, whereas the Cochrane review (25) uses data from the main publications from the same trial - Van der Ham et al. 2012a (26–31), 2012b (1,32–34) - which include a few more participants with undetermined GBS status. |

Table S2.02:Treatment effects (expectant management -v- immediate birth): respiratory distress syndrome

|  | GBS status | N RCTs | Odds ratio (95%CI) | *I* ^2^ | Model |
| --- | --- | --- | --- | --- | --- |
| Included RCTs | GBS+ only | 1 | 0.47 (0.09 to 2.56) | NA | NA |
| Included RCTs | All | 3 | 0.64 (0.47 to 0.89) | 0% | FE |
| **Cochrane review (base case)** | **All** | **5** | **0.67 (0.50 to 0.90)** | **0%** | **FE** |

## Caesarean birth

For probability of caesarean birth, we assume that the mother’s GBS status is likely to have minimal impact. Therefore, in our base case, we use data from the 5 RCTs pooled in the Cochrane review (25).

Figure S2.03: Treatment effects (expectant management -v- immediate birth): caesarean section

| 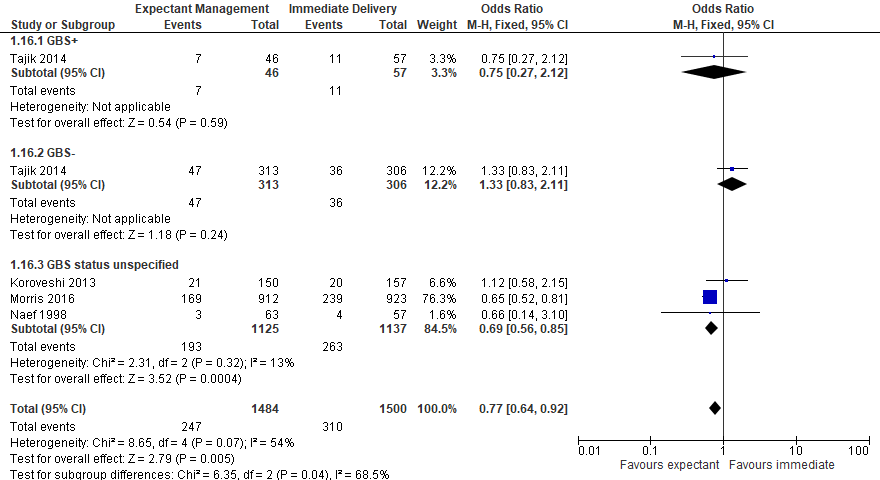 |
| --- |
| Note that the pooled total in this graph is very slightly different from the estimate from the Cochrane review we use in our model because it shows GBS-stratified results from Tajik et al. (1), to explore evidence for GBS-related heterogeneity, whereas the Cochrane review uses data from the main publications from the same trial - Van der Ham et al. 2012a (26–31), 2012b (1,32–34) - which include a few more participants with undetermined GBS status. |

Figure S2.03 shows a stratified forest plot for the analysis. There is a degree of heterogeneity between results, with the 1 exclusively GBS-negative datapoint appearing to show a different pattern. However, the pooled total is closely comparable with the estimate from the GBS-positive subgroup of the 1 RCT that stratifies results (1). Therefore, at the point estimate, it makes little difference which dataset we use, although uncertainty is obviously reduced in the bigger sample.

Table S2.03: Treatment effects (expectant management -v- immediate birth): caesarean birth

|  | GBS status | N RCTs | Odds ratio (95%CI) | *I* ^2^ | Model |
| --- | --- | --- | --- | --- | --- |
| Included RCTs | GBS+ only | 1 | 0.75 (0.27 to 2.12) | NA | NA |
| Included RCTs | All | 3 | 0.86 (0.50 to 1.48) | 73% | RE |
| **Cochrane review (base case)** | **All** | **5** | **0.78 (0.65 to 0.94)** | **62%** | **FE** |

# Derivation of model parameters – quality of life

## Impact of neonatal critical care

The model does not account for QALY loss due to the initial acute events, as the duration of these events is relatively short and there is no way of empirically quantifying HRQoL in affected neonates.

However, we assume when a newborn baby needs critical care, it is invariably an extremely stressful experience for the parents. Therefore, any mode of management that can increase or reduce the duration of NICU admission is likely to have an impact on their quality of life. We found no published information relating to the quality of life of parents of babies on NICU. Therefore, we have included an approximate estimate of the maternal impact of neonatal intensive care. We assume that the mother of a child in intensive care will be extremely anxious. We note that the EQ-5D utility value for an otherwise healthy person with extreme anxiety or depression is 0.414, which is 0.516 lower than the average for a woman in the UK aged 25–34 (35). This would give an annualised QALY decrement of 0.516, which equates to a loss of −0.001413 QALYs per day. The model therefore assumes that each day in NICU is associated with this level of QALY loss. As this figure lacks empirical foundation, we fitted a broad triangular distribution to vary this parameter in probabilistic analyses and tested the impact in deterministic sensitivity analysis.

The model does not account for QALY loss to the family in the event of neonatal death. A recent analysis by NICE’s Decision Support Unit (DSU) (36) examining how health-related quality of life has been modelled for carers found only 1 relevant analysis. This was a model submitted by the manufacturer of a technology undergoing highly specialised technology assessment that included a QALY loss seeking to quantify the impact of a child’s death ([NICE HST7](https://www.nice.org.uk/guidance/hst7/)) (37). However, this impact was not included in the company’s base case; it was a scenario analysis achieved by synthesising heterogeneous pieces of evidence that were of tenuous relevance to the decision problem. Accordingly, NICE’s decision-making committee considered the analysis did not accurately quantify the impact, and chose to consider this aspect of their decision problem in qualitative terms. Aside from this model, the DSU analysis found relatively little evidence from the wider literature on estimating the QALY impact on carers, and none regarding a QALY loss to the family in the event of child death.

Therefore, in the absence of a credible way to quantify the impact, our model does not estimate the QALY loss to the family in the event of neonatal death. We acknowledge that this is a limitation of the model. Further research is needed to estimate accurately the impacts on the family in instances of events such as neonatal death.

## Long-term consequences of infection / sepsis

We use the same HRQoL values for the long-term consequences of both meningitis and sepsis; that is, although we make use of evidence suggesting that the risk of sequelae is different for meningitis and sepsis, and the severity of impact also varies between the 2, the model treats, e.g., ‘moderate neurological impairment’ caused in either way as the same.

Previous analyses (3,4) have accounted for long-term neurological impairment using utility estimates from Oostenbrink et al. (36). This study used the EQ-5D to estimate HRQoL associated with permanent sequelae of meningitis. However, the valuations of each outcome were given by Dutch clinicians (rather than patients or carers, as NICE’s methods prefer) and do not explicitly relate to the outcomes modelled – for mild disability, previous authors have used Oostenbrink et al.’s value for deafness as a proxy; for moderate disability, they have relied on the category ‘mild mental retardation’; for severe disability, ‘epilepsy, mental retardation and leg paresis’. These factors make this source suboptimal, so we reserve it for a scenario analysis.

Instead, our base-case relies on values from a more recent UK cohort of extremely preterm babies followed up until 11 years of age (38). The valuations are from the children’s parents, and are based on the Health Utilities Index Mark 3 (HUI3) instrument. As this study also includes a contemporaneous control group, we can calculate utility multipliers directly; see Table S2.01. Despite our misgivings about the derivation of values from Oostenbrink et al.’s study, the multipliers for each category are relatively similar.

Table S2.01: Utility associated with neurodevelopmental disability following meningitis or sepsis

|  | N | Utility / disutility by level of impairment | | | |
| --- | --- | --- | --- | --- | --- |
|  |  | None | Mild | Moderate | Severe |
| Base case | | | | | |
| (38) | 196 | 0.959 (SE 0.008)^a^ | −0.179 (SE 0.042)^b^ | −0.298 (SE 0.055)^b^ | −0.558 (SE 0.084)^b^ |
|  |  |  | 0.813^c^ | 0.689^c^ | 0.418^c^ |
| Alternative value (scenario analysis) | | | | | |
| (36) | 28 | 1.000 | 0.810 (SD 0.150)^d^ | 0.620 (SD 0.110)^d^ | 0.470 (SD 0.250)^d^ |
| 1. Control group (N=135) of mainstream school classmates 2. Values are absolute disutilities compared with no impairment, estimated from multivariable regression adjusting for clinical and sociodemographic confounders 3. Equivalent utility multipliers 4. Published values are absolute utility estimates using EQ-5D; however, as they are the result of an exercise in which clinicians were asked to rate various sequelae alongside a ‘healthy’ state, they can be interpreted as relative to utility of 1; therefore, we can treat them as utility multipliers | | | | | |

## Long-term consequences of bronchopulmonary dyplasia

The model assumes no direct QALY loss due to RDS, for the same reasons we do not account for the immediate impact of infections. However, as previously described, the model simulates a proportion of babies with RDS will be categorised as having BPD, a proportion of whom will, in turn, experience lifelong sequela(e).

For the proportion of people experiencing asthma / wheezing, we draw our estimate of disutility from an extensive analysis of data from the English General Practice Patient Survey 2011–2012 (39), including 102,070 out of 906,578 (10.8%) respondents reporting ‘Asthma or long-term chest problem’. In a multivariable analysis adjusting for sociodemographic factors and the presence of many other conditions, the authors estimate the independent effect of asthma to be associated with a disutility of −0.058 (95%CI: −0.063 to −0.053) against a background expected utility value of 0.933 (95%CI 0.932 to 0.935) for people with no chronic health conditions. However, asthma is a common condition and, because we want to estimate the sequelae of BPD over and above what would be expected for people not experiencing it, we adjust general population utility to reflect the proportion of people who have asthma: 0.933 − 0.053 × 0.108 = 0.927. This gives us a final utility multiplier of (0.933 − 0.053) ÷ 0.927 = 0.944.

For neurodevelopmental sequelae of BPD, we use the same evidence we use for infection (see Table S2.01). However, the datasource we use to estimate the likelihood of BPD sequelae (14) distinguishes between ‘developmental delay’ and ‘neurological impairment’, whereas our utility values reflect a single, broader category incorporating the 2. Therefore, the model assumes that ‘developmental delay’ equates to ‘mild neurodevelopmental impairment’ (utility multiplier 0.813), ‘neurological impairment’ equates to ‘moderate neurodevelopmental impairment’ (utility multiplier 0.689), and experiencing both ‘developmental delay’ and ‘neurological impairment’ equates to ‘severe neurodevelopmental impairment’ (utility multiplier 0.418). As a sensitivity analysis, we use a weighted average of all 3 categories – weighted according to the proportions reported by Petrou et al. (38) (117/57/22 mild/moderate/severe) – for both outcomes.

## Consequences of caesarean birth for future pregnancies

The model assumes caesarean birth is associated with a negative impact on QALYs from an increased risk of ectopic pregnancy, miscarriage and stillbirth in future pregnancies.

The model assumes miscarriage is associated with an absolute decrement of 0.1 QALYs. This replicates the assumption used in NICE’s guideline on ectopic pregnancy and miscarriage ([NG126](https://www.nice.org.uk/guidance/ng126)) (21). However, it should be noted that there is no empirical basis to the value; rather, it was used as a starting-point for a range of sensitivity analyses in the absence of an evidence-based parameter. Similarly, we did not identify a suitable source for utility decrement of ectopic pregnancy, so we assume it has the same QALY impact as miscarriage, and test a broad range of values in sensitivity analysis.

For each stillbirth, the model subtracts an expected lifetime’s discounted QALYs to reflect the loss of a life (25.08 QALYs when discounted at 3.5% per year). While we acknowledge that this event will also have a profound impact on the child’s parents, we did not identify any suitable sources to help us quantify this effect. We therefore assume that any attempt to approximate the true impact would be inadequate, and it is better simply to note this as a limitation of our analysis.

As these parameters are less directly evidence-based than other model inputs, we subject them to extreme-value sensitivity analysis; see S5.1, below.

# Derivation of model parameters – costs

## Antenatal care

The categories of antenatal care the model accounts for are those enumerated by Lain et al. (40): inpatient admissions, day cases and outpatient appointments. Unit costs for these categories (taken from NHS Reference Costs 2016/17 (41) and subsequently inflated; see below) are shown in Table S3.01 and Table S3.02.

Table S3.01: Unit costs (2016/17) for antenatal care – inpatient admissions

| Code | Nonelective admissions | | | | Excess bed-days | | Average | | Weighted average per day |
| --- | --- | --- | --- | --- | --- | --- | --- | --- | --- |
|  | Mean (SE^a^) | Subm- issions | Epi- sodes | Mean LoS (d) | Mean (SE^a^) | N | Per episode | Per day |  |
| NZ17A^b^ | £1,953 (£45) | 213 | 1,747 | 2.47 | £425 (£21) | 1,486 | £2,314 | £698 | £677.58 |
| NZ17B^c^ | £1,719 (£26) | 394 | 7,651 | 2.40 | £512 (£13) | 4,841 | £2,043 | £673 |  |
| 1. Estimated from published interquartile range and number of submissions: SE = ([UQ−LQ] ÷ 1.349) ÷ √n, where 1.349 is 2 × the 0.75^th^ quantile of the standard normal distribution. 2. Ante-Natal False Labour, including Premature Rupture of Membranes, with CC Score 2+ 3. Ante-Natal False Labour, including Premature Rupture of Membranes, with CC Score 0-1 | | | | | | | | | |

Table S3.02: Unit costs (2016/17) for antenatal care – day cases and outpatient appointments

| Category | Code | Mean (SE^a^) | Subm- issions | Epi- sodes | Weighted average |
| --- | --- | --- | --- | --- | --- |
| Day cases | NZ17A^b^ | £292 (£52) | 8 | 52 | £278.03 |
|  | NZ17B^c^ | £278 (£4) | 31 | 1,877 |  |
| Outpatient appointments | WF01A^d^ (501 Obstetrics) | £120 (£5) | 134 | 1,539,008 | £120.20 |
| 1. Estimated from published interquartile range and number of submissions: SE = ([UQ−LQ] ÷ 1.349) ÷ √n, where 1.349 is 2 × the 0.75^th^ quantile of the standard normal distribution. 2. Ante-Natal False Labour, including Premature Rupture of Membranes, with CC Score 2+ 3. Ante-Natal False Labour, including Premature Rupture of Membranes, with CC Score 0-1 4. Non-Admitted Face-to-Face Attendance, Follow-up | | | | | |

We use these costs to value the resource-use observed in PPROMT (40), as shown in Table S3.03, which also shows values for the scenario analysis relying directly on total costs from the same publication. As expected, expectant management is associated with greater antenatal expenditure, with the difference between the 2 approaches amounting to somewhere in the region of £1,450–£1,650, depending on costing approach.

Table S3.03: Cost calculations for antenatal care

| Strategy | Resource-use – mean (SE) from Lain et al. (40) | | | Total costs | Inflated to 2018/19 |
| --- | --- | --- | --- | --- | --- |
|  | Inpatient days | Day cases | Outpatient appts |  |  |
| Base case – microcosting | | | | | |
| Immediate birth | 1.09 (0.05) | 0.09 (0.03) | 0.06 (0.02) | £770.79^a^ | £797.75 |
| Expectant management | 3.27 (0.14) | 0.49 (0.06) | 0.17 (0.03) | £2,372.35^a^ | £2,455.30 |
| Scenario analysis – total costs from Lain et al. (40) | | | | | |
| Immediate birth | – | – | – | £724.00**^b^** | £804.17 |
| Expectant management | – | – | – | £2,046.00^b^ | £2,272.56 |
| 1. Cost year = 2016/17 2. Cost year = 2011/12 | | | | | |

## Delivery costs

The costs associated with delivery are a simple function of the expected balance of caesarean and non-caesarean births. For the unit costs of non-caesarean birth, we use a weighted average of all vaginal (including instrumental) delivery codes in the NHS Reference Costs (41). This set comprises 30 HRGs: NZ30A–C, NZ31A–C, NZ32A–C, NZ33A–C, NZ34A–C, NZ40A–C, NZ41A–C, NZ42A–C, NZ43A–C and NZ44A–C. We include costs recorded under the following categories: elective (including excess bed-days), nonelective (including excess bed-days), nonelective short stay, day case, and community health services. In total, this amounts to 461,590 episodes across 210 category–HRG codes. For brevity, we do not reproduce each individual cost estimate here, but each is included in the model along with an estimate of its standard error (calculated as noted in Table S3.01); in probabilistic mode, the model calculates an average (weighted according to activity) of sampled values for all codes. The deterministic mean is £2,478.58 which, when uprated from 2016/17, equates to £2,565.25 in 2018/19 value.

Similarly, caesarean birth unit costs are calculated as a weighted average of values recorded under HRGs NZ50A–C (planned) and NZ51A–C (emergency), with elective (including excess bed-days), nonelective (including excess bed-days), nonelective short stays and day cases included. Table S3.04 shows the mean values derived in this way.

Table S3.04: Unit costs for caesarean births

| Type | Codes | Episodes | Mean (2016/17 values) | Inflated to 2018/19 |
| --- | --- | --- | --- | --- |
| Planned | NZ50A–C | 74,652 | £3,557.42 | £3,681.81 |
| Emergency | NZ51A–C | 97,979 | £4,780.59 | £4,947.76 |
| Planned + emergency |  | 172,631 | £4,251.65 | £4,400.32 |

For costing purposes, we split caesarean births into 2 categories: those that would be expected, and excess events arising as a result of the chosen mode of management. The evidence we use in the model suggests that immediate birth is most likely to be associated with more caesareans though, in any given iteration of the probabilistic model, it is possible that an OR>1 will be sampled, implying expectant management leads to more caesareans. For the expected events (the caesarean births that would have happened one way or another), we assume the procedures are a mixture of planned and emergency procedures, in the same proportions observed in the general population. For the excess events (the caesarean births that result from the chosen mode of managing the PPROM), we assume all procedures would be coded as emergencies. Table S3.05 shows the base-case calculations, alongside values for the scenario analysis relying directly on total costs from Lain et al. (40). The 2 approaches reach similar conclusions, with immediate birth associated with a small increase in costs in the range £133–£212.

Table S3.05: Cost calculations for delivery

| Strategy | Proportions | | | Total costs | Inflated to 2018/19 |
| --- | --- | --- | --- | --- | --- |
|  | Expected caesareans (planned & emergency) | Excess caesareans (emergency) | Non- caesareans |  |  |
| Base case – microcosting | | | | | |
| Immediate birth | 0.301 | 0.056 | 0.643 | £3,140.75^a^ | £3,250.58 |
| Expectant management | 0.301 | – | 0.699 | £3,012.42^a^ | £3,117.76 |
| Scenario analysis – total costs from Lain et al. (40) | | | | | |
| Immediate birth | – | – | – | £2,867.00^b^ | £3,184.48 |
| Expectant management | – | – | – | £2,676.00^b^ | £2,972.33 |
| 1. Cost year = 2016/17 2. Cost year = 2011/12 | | | | | |

## Neonatal costs

The costs associated with hospital care for the newborn baby will be substantially affected by the incidence of infections. As previously noted, we decided when it comes to infections, we should only use evidence from trial subgroups that reflect our population of interest – that is, women with prior detection of GBS. As a result, the expected rate of neonatal infections in our model is quite different from that observed in the overall trial populations, both in absolute and relative terms. For this reason, it would not be appropriate to use resource-use or total cost estimates from Lain et al. (40) directly, as they represent the rate of infections observed in the overall RCT population, regardless of the mothers’ GBS status.

To address this problem, we adopt a relatively simple 4-stage approach that aims to disaggregate costs directly associated with infections and other neonatal care costs. (A) We calculate the costs observed in the full PPROMT population (40), in the same way as for the previous categories of perinatal costs. (B) We estimate the additional costs associated with an average neonatal infection, compared with a baby who does not experience this event. (C) We multiply the cost by the infection rate observed in the full trial population of PPROMT, and deduct those costs from the estimate calculated in step (A), to provide an estimate of the resource-use and costs that would be expected if none of the neonates had experienced an infection. (D) We multiply our estimate of infection costs by the rates of infections we expect in each modelled arm of our GBS+ population, and add those back on to our estimate of costs without infections, to provide an estimate of the resource-use and costs that corresponds to the rate of infections in the model.

Table S3.06 shows the daily costs we use for all these calculations. Where we require a unit cost for critical care without further specification as to level of support, we use an activity-weighted average of codes XA01Z–XA04Z. This amounts to £721.44 per day.

Table S3.06: Unit costs (per day) for neonatal care

| Code | Submissions | Days | Mean cost per day (SE^a^)^b^ | Inflated to 2018/19 |
| --- | --- | --- | --- | --- |
| XA01Z^c^ | 129 | 159,664 | £1,295 (£34) | £1,340 |
| XA02Z^d^ | 129 | 183,555 | £897 (£18) | £929 |
| XA03Z^e^ | 129 | 535,683 | £577 (£15) | £597 |
| XA04Z^f^ | 106 | 152,758 | £418 (£19) | £432 |
| XA05Z^g^ | 96 | 61,167 | £423 (£19) | £438 |
| 1. Estimated from published interquartile range and number of submissions: SE = ([UQ−LQ] ÷ 1.349) ÷ √n, where 1.349 is 2 × the 0.75^th^ quantile of the standard normal distribution. 2. Cost year = 2016/17 3. Neonatal Critical Care, Intensive Care 4. Neonatal Critical Care, High Dependency 5. Neonatal Critical Care, Special Care, without External Carer 6. Neonatal Critical Care, Special Care, with External Carer 7. Neonatal Critical Care, Normal Care | | | | |

Table S3.07 shows the calculation of neonatal costs for the (step (A) as explained above).

There is a larger difference between the 2 approaches than in previous categories. We speculate this may be because we have a single cost category, costed as a weighted average of codes XA01Z–XA04Z, for all days of critical care. However, it is plausible that the immediate birth arm, which had a greater proportion of critical care and a greater duration of critical care than the expectant management arm, also featured a greater proportion of the most intensive, expensive critical care within that category. It is not possible for us to account for this using the data available to us.

Table S3.07: Cost calculations for neonatal care as observed in overall trial population (regardless of mothers’ GBS status)

| Strategy | Critical care | | | | Mean overall LoS in hospital – d (SE) | Postnatal ward  – d^c^ | Total cost | Inflated to 2018/19 |
| --- | --- | --- | --- | --- | --- | --- | --- | --- |
|  | % admitted | Mean stay – d (SE)^a^ | Mean stay per patient – d^b^ | Cost |  |  |  |  |
| Base case – microcosting | | | | | | | |  |
| Immediate | 68.5% (631/921) | 8.9 (0.3) | 6.1 | £4,538 | 7.4 (0.2) | 1.3 | £4,953^d^ | £5,126 |
| Expectant | 59.1% (537/908) | 7.8 (0.3) | 4.6 | £3,453 | 6.4 (0.2) | 1.8 | £4,101^d^ | £4,244 |
| Scenario analysis – total costs from Lain et al. (40) | | | | | | | |  |
| Immediate | – | – | – | – | – | – | £5,261^e^ | £5,844 |
| Expectant | – | – | – | – | – | – | £4,022^e^ | £4,467 |
| 1. Mean stay among those admitted to critical care 2. Mean stay in critical care for the average patient (i.e. probability of admission × mean stay among those admitted) 3. Overall LoS minus critical care 4. Cost year = 2016/17 5. Cost year = 2011/12 | | | | | | | | |

Table S3.08 sets out the calculations for step (B) of our process: estimating the excess resource-use and costs associated with neonatal infections. Our estimates are based on a prospective cohort study of infants with GBS disease in England (6). This study provides detailed data on resource-use for 138 infants (<90 days) experiencing early- or late-onset GBS infection, compared with 305 contemporaneous controls (matched for birthweight) who had no clinically evident infections. This is an ideal datasource for our analysis, with the single shortcoming that it reports relatively historical practice (2000–03).

Table S3.08: Cost calculations for infections

| Outcome | Days – mean (SE) from Schroeder et al. (6) | | | | Total costs | Inflated to 2018/19 |
| --- | --- | --- | --- | --- | --- | --- |
|  | NICU | HDU | SCU | Postnatal |  |  |
| Base case – microcosting | | | | | | |
| Infections | 3.8 (0.9) | 3.8 (0.6) | 10.4 (1.1) | 0.5 (0.3) | £14,174^a^ | £14,669 |
| Controls | 1.9 (0.5) | 1.4 (0.3) | 4.6 (0.6) | 2.0 (0.1) | £7,054^a^ | £7,301 |
| Difference | 1.9 | 2.4 | 5.8 | −1.5 | £7,120^a^ | £7,369 |
| Scenario analysis – total costs from Schroeder et al. (6) | | | | | | |
| Difference | – | – | – | – | £5,209 (£1,286^b^)^c^ | £6,543 |
| 1. Cost year = 2016/17 2. Calculated from published bootstrapped 95% confidence interval (£2,843.3 to £7,885.80) 3. Cost year = 2003 | | | | | | |

The final calculations, using the outputs of the 2 previous steps and performing steps (C) and (D), appear in Table S3.09. The inclusion of expected costs of infection attenuates the advantage expectant management would otherwise have over immediate birth in this area. Nevertheless, immediate birth, with its higher proportion of premature babies, remains the more expensive approach, with an additional cost per baby of £237–£731, depending on the approach we use.

Table S3.09: Final cost calculations for neonatal care

| Strategy | Whole RCT population | | | | GBS+ population | | |
| --- | --- | --- | --- | --- | --- | --- | --- |
|  | Total costs | Observed infections | Deduct cost of infections | Costs with no infections | Expected infections | Costs of infections | Final estimate |
| Base case – microcosting | | | | | | | |
| Immediate | £5,126 | 2.5% (23/923) | −£184 | £4,942 | 5.8% | £426 | £5,368 |
| Expectant | £4,244 | 3.2% (29/912) | −£234 | £4,010 | 15.2% | £1,121 | £5,131 |
| Scenario analysis – total costs from Lain et al. (40) | | | | | | | |
| Immediate | £5,844 | 2.5% (23/923) | −£184 | £5,660 | 5.8% | £426 | £6,086 |
| Expectant | £4,467 | 3.2% (29/912) | −£234 | £4,233 | 15.2% | £1,121 | £5,354 |

The other major neonatal event our model accounts for (in terms of outcomes) is RDS. However, it is not necessary to cost these events separately in a similar way to infections. Our assumption that a mother’s GBS status is unlikely to have a meaningful effect on the likelihood of RDS, and the data bear this out as regards both absolute and relative event-rates. Notably, the estimates from PPROMT (2) are typical of the overall dataset and closely comparable with the values from the 1 GBS+ subgroup for which we have data (1). Therefore, we have some confidence that the resource-use data from the same trial (40) reflects a level of RDS that closely corresponds to the expectation in our modelled population.

## Total perinatal costs

Table S3.10 summarises the results of calculations across all 3 categories of perinatal care. Expectant management appears to be the more expensive approach, mostly as a result of increased antenatal costs. The size of the estimated difference depends on costing approach, with the largest discrepancy arising in neonatal care costs, as discussed above.

Table S3.10: Total perinatal costs

| Category | Immediate birth | Expectant management | Difference |
| --- | --- | --- | --- |
| Base case – microcosting | | | |
| Antenatal | £797.75 | £2,455.30 | −£1,657.56 |
| Delivery | £3,250.58 | £3,117.76 | £132.82 |
| Neonatal | £5,367.93 | £5,130.94 | £236.99 |
| Total | £9,416.25 | £10,704.00 | −£1,287.74 |
| Scenario analysis – total costs from Lain et al. (6) | | | |
| Antenatal | £804.17 | £2,272.56 | −£1,468.39 |
| Delivery | £3,184.48 | £2,972.33 | £212.15 |
| Neonatal | £6,085.73 | £5,354.39 | £731.34 |
| Total | £10,074.38 | £10,599.28 | −£524.90 |

## Long-term morbidity – cost per year

### Costs associated with disability due to infection

As detailed previously, we account for lifelong neurodevelopmental morbidity secondary to neonatal infection. The model subdivides cases into mild, moderate and severe impairment, with the relative prevalence of each depending on whether the person experienced meningitis or sepsis as a neonate.

To estimate the costs with which these outcomes are associated, we rely on publications from the EPICure longitudinal study of premature babies in the UK and Ireland - Mangham et al. (38). The clear strength of these sources is that they provide detailed, UK-specific data on NHS, PSS and wider public sector costs associated with neurodevelopmental disability in a cohort followed up for over a decade, with contemporaneous controls. Their major limitation, from our perspective, is that the cohort in question were all born at 20–25 completed weeks’ gestation, much more prematurely than our population of interest. However, although the incidence of neurodevelopmental disability is higher in this population (and the proportion experiencing more severe impairment may also be raised), there is no reason to believe that children classified as having mild, moderate or severe impairment will have meaningfully different prospects to those experiencing mild, moderate or severe impairment in the less premature population in which we are interested. This evidence has been used to quantify the impact of neonatal insults in several economic evaluations, including previous NICE guidance ([Specialist neonatal respiratory care for babies born preterm [NG124]](https://www.nice.org.uk/guidance/ng124/)) (42) and published studies pertaining to neonatal infection (9).

Alongside inflating the reported costs to present-day values, we also had to perform some calculations to estimate NHS+PSS costs and those associated with ‘broader public sector’ activity (this includes the costs of state-funded education). We do this by estimating a ratio between the 2 categories and applying it in all cases; this approach is similar to that adopted in NG124 (42). In one of the publications (38), the authors note that severe neurodevelopmental impairment resulted in an average unadjusted increase of £1,085 in NHS+PSS costs, and £8,797 in public sector costs. Although the authors do not provide a similar breakdown across all categories of impairment (or give an estimate of values adjusted for other clinical and sociodemographic factors, as they helpfully do for their total costs), we assume that the same ratio between NHS+PSS and other public sector costs applies throughout – that is, 1:8.1; equivalent to saying that NHS+PSS costs make up 11% of additional public expenditure, with other public sector costs (education) accounting for the remainder. Table S3.11 provides details.

Table S3.11: Annual costs associated with neurodevelopmental impairment

| Category | Degree of neurodevelopmental disability | | | |
| --- | --- | --- | --- | --- |
|  | None | Mild | Moderate | Severe |
| Preschool - source: (43) | | | | |
| Total absolute costs | £315.00^a^ | £611.00^a^ | £660.00^a^ | £1,206.00^a^ |
| Additional total costs of disability | – | £296.00 | £345.00 | £891.00 |
| **Inflated from 2005/06 to 2018/19** | **–** | **£347.88** | **£405.46** | **£1,047.16** |
| Additional NHS+PSS costs of disability | – | £296.00^b^ | £345.00^b^ | £891.00^b^ |
| Inflated from 2005/06 to 2018/19 | – | £347.88 | £405.46 | £1,047.16 |
| Additional public sector costs of disability | – | –^b^ | –^b^ | –^b^ |
| Primary school - source: (43) | | | | |
| Total absolute costs | £3,467.00^a^ | £3,763.00^a^ | £4,814.00^a^ | £12,389.00^a^ |
| Additional total costs of disability | – | £296.00 | £1,347.00 | £8,922.00 |
| **Inflated from 2005/06 to 2018/19** | **–** | **£347.88** | **£1,583.08** | **£10,485.67** |
| Additional NHS+PSS costs of disability | – | £32.50^c,d^ | £147.89^c,d^ | £979.60^c,d^ |
| Inflated from 2005/06 to 2018/19 | – | £38.20 | £173.81 | £1,151.28 |
| Additional public sector costs of disability | – | £263.50^c,d^ | £1,199.11^c,d^ | £7,942.40^c,d^ |
| Age 11 onwards - source: (38) | | | | |
| Total absolute costs | NR | NR | NR | NR |
| Additional total costs of disability | – | £3,612.17^e^ | £5,969.27^e^ | £9,701.66^e^ |
| **Inflated from 2006/07 to 2018/19** | **–** | **£4,537.54** | **£7,498.50** | **£12,187.07** |
| Additional NHS+PSS costs of disability | – | £396.60^a,f^ | £655.40^a,f^ | £1,065.20^a,f^ |
| Inflated from 2006/07 to 2018/19 | – | £498.20 | £823.30 | £1,338.09 |
| Additional public sector costs of disability | – | £3,215.57^c,g^ | £5,313.87^c,g^ | £8,636.46^c,g^ |
| 1. These are the data directly reported in the publications 2. Although it is not entirely clear, it appears that the authors only include education in the category of ‘broader public sector’ costs; therefore, we assume that 100% of total costs for preschool children relate to NHS+PSS expenditure 3. We assume that the ratio between NHS+PSS and other public sector costs is 1:8.11 (based on information in Petrou et al. 2013***; see text) 4. We use the assumed ratio to estimate the split between NHS+PSS and other public sector costs, from the published total amount for the 2 categories 5. Sum of published NHS+PSS costs and estimated additional public sector costs 6. Estimates from a multivariable model adjusting for various clinical and sociodemographic factors, in an attempt to isolate the independent impact of neurodevelopmental impairment 7. We use the assumed ratio to estimate additional public sector costs, from the published NHS+PSS costs | | | | |

Previous economic evaluations simulating the consequences of neonatal infection (3,4) have used long-term cost estimates that can be traced to a model of meningitis vaccination (44). Those authors assumed 10% of meningitis survivors would require lifelong, full-time residential care and the remainder would accrue additional healthcare costs £500 per year, though no empirical basis is provided. While we are confident that our base-case costing represents a more evidence-based method, we replicate the older approach in a sensitivity analysis, to see if the methods adopted by earlier modellers have a meaningful effect on results. The equivalent numbers are £79,013.93 per year for severe impairment – derived from the Adult Social Care Activity and Finance Report, England – 2018–19 (45) – and £831.90 per year for mild and moderate disability (£500 inflated from 1999/2000 to 2018/19).

### Costs associated with disability due to BPD

As for utilities, we assume that ‘developmental delay’ as a consequence of BPD equates to ‘mild neurodevelopmental impairment’, ‘neurological impairment’ equates to ‘moderate neurodevelopmental impairment’, and a combination of the 2 equates to ‘severe neurodevelopmental impairment’ and use the appropriate annual values from Table S3.11. As a sensitivity analysis, we use a weighted average of all 3 categories – weighted according to the proportions reported by Petrou et al. (117/57/22 mild/moderate/severe) – for both outcomes (38).

Following NICE’s guideline on asthma ([NG80](https://www.nice.org.uk/guidance/ng80)) (46), we use a weighted average of costs across different levels of control and frequency of exacerbations (47) to estimate an annual cost for asthma. When inflated to 2018/19 values, this amounts to £330.50 per year. As for our quality of life estimate, we adjust this value to reflect the proportion of people who would experience asthma even without BPD: this means we estimate a year of asthma secondary to BPD costs £294.81 over and above asthma costs for an average member of the population.

## Consequences of caesarean birth for future pregnancies

Our approach to estimating the costs of miscarriage is substantially based on the methods used by the National Guideline Alliance (NGA) in work commissioned by the Human Fertilisation and Embryology Authority and others (48). We calculate the average cost of a miscarriage requiring hospital care (Table S4.01) and apply that to the proportion of events that receive that level of care. Here, we diverge from the NGA’s estimate. They assume only 20% of miscarriages fall into this category, based on a suggestion that there are up to 250,000 miscarriages per year in the UK, compared with around 50,000 episodes in the NHS Reference Costs. We agree that a little under 50,000 episodes is a reasonable numerator (see Table S4.01); however, we believe that, for our purposes, 250,000 is an overestimate of the total number of events we should account for. This is partially because it relates to the whole of the UK (whereas NHS reference costs cover England alone). Moreover, while we do not doubt that it may be an accurate estimate of the total number of miscarriages per year including those that do not come to the attention of medical services or even the woman herself, we need to estimate those incurring medical costs. Evidence used elsewhere in our analysis suggests that 12.8% of pregnancies result in miscarriage that is recorded in medical records (23). Applying this proportion to the number of live births in England (603,766 in 2018/19) suggests that we would expect around 90,000 medically recorded miscarriages. Therefore, to avoid the appearance of spurious precision, we make the simple assumption that half of miscarriages coming to medical attention require hospital care. We then adopt the NGA’s assumption that all miscarriages require an average of 1 GP appointment – costed at £39.23 each, per the Unit Costs of Health and Social Care, (49). This gives us a final estimate of £666.47 × 0.5 + £39.23 = £372.47 per simulated event.

Ectopic pregnancy

The developers of NICE’s guidance on ectopic pregnancy and miscarriage ([NG126](https://www.nice.org.uk/guidance/ng126)) (21) undertook detailed costing for 3 ways of managing ectopic pregnancies: salpingectomy, salpingotomy and medical management. They estimated average costs of £1,608, £2,205 and £1,432, respectively. We then required an estimate of the relative frequency of each, in order to arrive at a weighted average for the typical ectopic pregnancy. However, we were unable to find any suitable data in the literature or in publicly available routine data. Therefore, we obtained a dedicated extract of Hospital Episode Statistics (HES), detailing all episodes under ICD-10 code O00. This showed that a substantial majority of activity was recorded under 11 codes: 5 indicate that salpingectomy was the major procedure in the episode (Q231, Q233, Q234, Q242, Q259; 6,880 episodes); 1 relates to salpingotomy (Q304; 71 episodes); and 3 show that no invasive procedure was carried out, suggesting medical management only (No procedure, Q555, X373; 2,449 episodes). The remaining 2 codes (Q111, Q311) relate to aspiration of products of conception, for which we have no cost estimate; however, this represents a small volume of cases (<300 total episodes), so we exclude them from calculations. We are left with a 0.732 : 0.008 : 0.261 weighting for salpingectomy, salpingotomy and medical management; applying this gives us a mean cost of £1,566.66 which, when inflated to 2018/19 value, amounts to £1,776.68. This is the cost we apply for all additional ectopic pregnancies arising in future pregnancies.

Stillbirth

Following NICE’s guideline on Intrapartum care for women with existing medical conditions or obstetric complications and their babies ([NG121](https://www.nice.org.uk/guidance/ng121)) (50), we obtain our estimate of the costs of stillbirth from a dedicated costing study (51). This suggests that an average stillbirth is associated with healthcare costs of £4,191.00; when inflated to 2018/19 value, this becomes £4,527.47.

Table S4.01: Unit costs for miscarriages requiring hospital treatment

| Categories and codes | Submissions | Episodes | Mean (SE^a^) |
| --- | --- | --- | --- |
| Nonelective | | | |
| MB08A | 203 | 1,025 | £2,034.51 (£55.34) |
| MB08B | 363 | 3,495 | £1,641.42 (£25.77) |
| Nonelective excess bed-days | | | |
| MB08A | 27 | 274 | £427.27 (£11.37) |
| MB08B | 208 | 1,480 | £607.04 (£13.87) |
| Nonelective total | | | |
| MB08A |  |  | £2,148.72 |
| MB08B |  |  | £1,898.48 |
| Elective | | | |
| MB08A | 29 | 38 | £2,082.31 (£262.98) |
| MB08B | 114 | 882 | £1,011.10 (£70.68) |
| Elective excess bed-days | | | |
| MB08A | 3 | 8 | £279.47 (£0.00^b^) |
| MB08B | 9 | 41 | £157.45 (£19.21) |
| Elective total | | | |
| MB08A |  |  | £2,141.15 |
| MB08B |  |  | £1,018.42 |
| Nonelective short-stay | | | |
| MB08A | 156 | 317 | £859.99 (£28.43) |
| MB08B | 648 | 39,204 | £497.77 (£8.64) |
| Day case | | | |
| MB08A | 5 | 7 | £584.16 (£248.72) |
| MB08B | 146 | 2,363 | £383.85 (£21.43) |
| Regular admission | | | |
| MB08B | 8 | 66 | £91.01 (£0.00) |
| Overall total | | | |
| MB08A |  | 1,387 | £1,846.08 |
| MB08B |  | 46,010 | £607.72 |
| Weighted average |  | 47,397 | £643.95 |
| Inflated from 2016/17 to 2018/19 |  |  | £666.47 |
| MB08A Threatened or Spontaneous Miscarriage, with Interventions  MB08B Threatened or Spontaneous Miscarriage, without Interventions   1. Estimated from published interquartile range and number of submissions: SE = ([UQ−LQ] ÷ 1.349) ÷ √n, where 1.349 is 2 × the 0.75^th^ quantile of the standard normal distribution. 2. SE unavailable because IQR=0 owing to low volume of activity | | | |

# Supplementary results

## One-way sensitivity analysis

Figure S5.01: One-way sensitivity analysis – tornado diagram

|  |
| --- |
| 30 most influential parameters shown. Positive incremental net health benefit implies immediate birth is the preferred option (i.e. it would be associated with an ICER of £20,000/QALY or better compared with expectant management) |

As noted in S3.4, above, we base our inputs estimating the quality-of-life impact of complications of future pregnancies on broad assumptions in the absence of relevant evidence. It can be seen from Figure S5.01 that these parameters are not among the 30 most influential in our model. However, we performed additional, extreme-value analyses to check the impact of these parameters on model results. When we set all 3 parameters (QALY-loss associated with miscarriage, ectopic pregnancy and stillbirth) to implausibly high values (10 QALYs lost per miscarriage or ectopic pregnancy; 50 QALYs lost per stillbirth), the incremental QALYs predicted for immediate birth, compared with expectant management, fell from 0.333 to 0.321. When we set all 3 parameters to 0 (implying these events have no quality-of-life impact at all), they rose to 0.335. These analyses show that, although we do not have an evidence-based estimate of these parameters, any value they could plausibly take would not meaningfully affect our decision uncertainty. This is because the choice between immediate delivery and expectant management has a small effect on probability of caesarean birth which, in turn, has a small effect on the likelihood of complications in future pregnancies (and, especially in the case of stillbirth, the complications themselves are relatively rare). As illustrated in figure 2, in the main text, the QALY impact of future pregnancies is dwarfed by the impact of other events, most notably neonatal infections.

## Threshold analysis

Figure S5.02: One-way sensitivity analysis – odds ratio for infection

|  |
| --- |
|  |
| Lower panel shows magnified section of upper panel.  Positive incremental net health benefit – when the red line is above the horizontal axis – implies immediate birth is the preferred option (i.e. it would be associated with an ICER of £20,000/QALY or better compared with expectant management) |

## Additional probabilistic sensitivity analyses

Below, we depict the results of probabilistic sensitivity analyses (1,000 iterations) where we hold a parameter of particular interest at one end of its uncertainty interval while varying all others across their probabilistic range in the usual way.

Figure S5.03: Probabilistic sensitivity analysis when we allow all parameters to vary except odds ratio for infection, which is fixed at its lower 95% confidence limit (0.33)

|  |
| --- |
|  |
| Above – cost-utility scatterplot. Below – cost-effectiveness acceptability curve. Bold line in CEAC shows cost-effectiveness acceptability frontier (CEAF). |

Figure S5.04: Probabilistic sensitivity analysis when we allow all parameters to vary except odds ratio for infection, which is fixed at its upper 95% confidence limit (26.19)

|  |
| --- |
|  |
| Above – cost-utility scatterplot. Below – cost-effectiveness acceptability curve. Bold line in CEAC shows cost-effectiveness acceptability frontier (CEAF). |

Figure S5.05: Probabilistic sensitivity analysis when we allow all parameters to vary except baseline probability of infection, which is fixed at its lower 95% confidence limit (0.063)

|  |
| --- |
|  |
| Above – cost-utility scatterplot. Below – cost-effectiveness acceptability curve. Bold line in CEAC shows cost-effectiveness acceptability frontier (CEAF). |

Figure S5.06: Probabilistic sensitivity analysis when we allow all parameters to vary except baseline probability of infection, which is fixed at its upper 95% confidence limit (0.289)

|  |
| --- |
|  |
| Above – cost-utility scatterplot. Below – cost-effectiveness acceptability curve. Bold line in CEAC shows cost-effectiveness acceptability frontier (CEAF). |

# References

1. Tajik P, van der Ham DP, Zafarmand MH, Hof MHP, Morris J, Franssen MTM, et al. Using vaginal Group B Streptococcus colonisation in women with preterm premature rupture of membranes to guide the decision for immediate delivery: a secondary analysis of the PPROMEXIL trials. BJOG An Int J Obstet Gynaecol [Internet]. 2014 Sep 1;121(10):1263–72. Available from: https://doi.org/10.1111/1471-0528.12889

2. Morris JM, Roberts CL, Bowen JR, Patterson JA, Bond DM, Algert CS, et al. Immediate delivery compared with expectant management after preterm pre-labour rupture of the membranes close to term (PPROMT trial): a randomised controlled trial. Lancet [Internet]. 2016 Jan 30;387(10017):444–52. Available from: https://doi.org/10.1016/S0140-6736(15)00724-2

3. Colbourn T, Asseburg C, Bojke L, Philips Z, Claxton K, Ades AE, et al. Prenatal screening and treatment strategies to prevent group B streptococcal and other bacterial infections in early infancy: cost-effectiveness and expected value of information analyses. Health Technol Assess. 2007;11(29):1–226.

4. National Institute for Health and Care Excellence. Antibiotics for early-onset neonatal infection: antibiotics for the prevention and treatment of early-onset neonatal infection [Internet]. NICE. London: National Institute for Health and Care Excellence (NICE); 2012. Available from: https://www.nice.org.uk/guidance/ng195/evidence/full-guideline-pdf-9078467006

5. O’Sullivan CP, Lamagni T, Patel D, Efstratiou A, Cunney R, Meehan M, et al. Group B streptococcal disease in UK and Irish infants younger than 90 days. Lancet Infect Dis [Internet]. 2019 Jan 1;19(1):83–90. Available from: https://doi.org/10.1016/S1473-3099(18)30555-3

6. Schroeder E-A, Petrou S, Balfour G, Edamma O, Heath PT, Group on behalf of the HPAGBSW. The economic costs of Group B Streptococcus (GBS) disease: prospective cohort study of infants with GBS disease in England. Eur J Heal Econ [Internet]. 2008;10(3):275. Available from: https://doi.org/10.1007/s10198-008-0131-4

7. Okike IO, Johnson AP, Henderson KL, Blackburn RM, Muller-Pebody B, Ladhani SN, et al. Incidence, Etiology, and Outcome of Bacterial Meningitis in Infants Aged <90 Days in the United Kingdom and Republic of Ireland: Prospective, Enhanced, National Population-Based Surveillance. Clin Infect Dis [Internet]. 2014 Nov 15;59(10):e150–7. Available from: https://doi.org/10.1093/cid/ciu514

8. National Institute for Health and Care Excellence. Overview | Neonatal infection (early onset): antibiotics for prevention and treatment | Guidance | NICE [Internet]. NICE. 2012 [cited 2021 Mar 28]. Available from: https://www.nice.org.uk/guidance/cg149

9. Grosso A, Neves de Faria RI, Bojke L, Donohue C, Fraser CI, Harron KL, et al. Cost-effectiveness of strategies preventing late-onset infection in preterm infants. Arch Dis Child [Internet]. 2020 May 1;105(5):452 LP – 457. Available from: http://adc.bmj.com/content/105/5/452.abstract

10. Office for National Statistics. National life tables, UK: 2016 to 2018 - Office for National Statistics [Internet]. ONS. 2019 [cited 2021 Mar 28]. Available from: https://www.ons.gov.uk/releases/nationallifetablesuk2016to2018

11. Reid SM, Carlin JB, Reddihough DS. Survival of individuals with cerebral palsy born in Victoria, Australia, between 1970 and 2004. Dev Med Child Neurol [Internet]. 2012 Apr [cited 2021 Mar 28];54(4):353–60. Available from: https://pubmed.ncbi.nlm.nih.gov/22329739/

12. Horbar J, Carpenter J, Kenny M eds. Vermont Oxford Network 2002 Very Low Birth Weight Database Summary. Burlington, VT: Vermont Oxford Network; 2003.

13. Zysman-Colman Z, Tremblay Msc GM, Landry JS. Bronchopulmonary dysplasia-trends over three decades. Paediatr Child Heal [Internet]. 2013 Feb [cited 2021 Apr 1];18(2):90. Available from: https://academic.oup.com/pch/article/18/2/86/2647035

14. Landry JS, Chan T, Lands L, Menzies D. Long-term impact of bronchopulmonary dysplasia on pulmonary function. Can Respir J [Internet]. 2011;18(5):265–70. Available from: https://pubmed.ncbi.nlm.nih.gov/21969927

15. NHS Digital. NHS Maternity Statistics, England 2018-19 [PAS] - NHS Digital [Internet]. NHS Maternity Statistics. 2019 [cited 2021 Mar 30]. Available from: https://digital.nhs.uk/data-and-information/publications/statistical/nhs-maternity-statistics/2018-19

16. National Institute for Health and Care Excellence. Neonatal infection: antibiotics for prevention and treatment [Internet]. NICE. 2021. Available from: https://www.nice.org.uk/guidance/ng195

17. Office for National Statistics. Childbearing for women born in different years, England and Wales - Office for National Statistics [Internet]. ONS. 2019. Available from: https://www.ons.gov.uk/peoplepopulationandcommunity/birthsdeathsandmarriages/conceptionandfertilityrates/bulletins/childbearingforwomenbornindifferentyearsenglandandwales/2018

18. Office for National Statistics. Births by parents’ characteristics - Office for National Statistics [Internet]. ONS. 2019 [cited 2021 May 20]. Available from: https://www.ons.gov.uk/peoplepopulationandcommunity/birthsdeathsandmarriages/livebirths/datasets/birthsbyparentscharacteristics

19. NMPA Project Team. National Maternity and Perinatal Audit: Clinical Report 2019. Based on births in NHS maternity services between 1 April 2016 and 31 March 2017. [Internet]. London; 2019. Available from: https://maternityaudit.org.uk/FilesUploaded/NMPA Clinical Report 2019.pdf

20. Keag OE, Norman JE, Stock SJ. Long-term risks and benefits associated with cesarean delivery for mother, baby, and subsequent pregnancies: Systematic review and meta-analysis. PLOS Med [Internet]. 2018 Jan 23;15(1):e1002494. Available from: https://doi.org/10.1371/journal.pmed.1002494

21. National Institute for Health and Care Excellence. Ectopic pregnancy and miscarriage: diagnosis and initial management [Internet]. NICE. 2019. Available from: https://www.nice.org.uk/guidance/ng126

22. Lewis G (ed). Saving Mothers’ Lives: reviewing maternal deaths to make motherhood safer 2003-2005. The Seventh Report on Confidential Enquiries into Maternal Deaths in the United Kingdom. [Internet]. London; 2007. Available from: https://www.publichealth.hscni.net/publications/saving-mothers-lives-2003-2005

23. Magnus MC, Wilcox AJ, Morken N-H, Weinberg CR, Håberg SE. Role of maternal age and pregnancy history in risk of miscarriage: prospective register based study. BMJ [Internet]. 2019 Mar 20;364:l869. Available from: http://www.bmj.com/content/364/bmj.l869.abstract

24. Office for National Statistics. Child and infant mortality in England and Wales - Office for National Statistics [Internet]. ONS. 2019 [cited 2021 May 20]. Available from: https://www.ons.gov.uk/peoplepopulationandcommunity/birthsdeathsandmarriages/deaths/bulletins/childhoodinfantandperinatalmortalityinenglandandwales/2017

25. Bond DM, Middleton P, Levett KM, van der Ham DP, Crowther CA, Buchanan SL, et al. Planned early birth versus expectant management for women with preterm prelabour rupture of membranes prior to 37 weeks’ gestation for improving pregnancy outcome. Cochrane Database Syst Rev [Internet]. 2017;(3). Available from: https://doi.org//10.1002/14651858.CD004735.pub4

26. Mol B, Vijgen S, Opmeer B, Bijlenga D, Akerboom B. Economic analysis of induction of labor versus expectant management in women with preterm prelabor rupture of membranes between 34 and 37 weeks (PPROMEXIL trial ISRCTN29313500). Am J Obstet Gynecol [Internet]. 2011 [cited 2021 May 27];204(1 Suppl):S336. Available from: https://www.narcis.nl/publication/RecordID/oai:openaccess.leidenuniv.nl:1887%2F98561

27. van der Ham DP, Vijgen SMC, Nijhuis JG, van Beek JJ, Opmeer BC, Mulder ALM, et al. Induction of labor versus expectant management in women with preterm prelabor rupture of membranes between 34 and 37 weeks: A randomized controlled trial. PLoS Med [Internet]. 2012 Apr [cited 2021 May 27];9(4):e1001208. Available from: www.plosmedicine.org

28. van der Ham DP, Nijhuis JG, Mol BWJ, van Beek JJ, Opmeer BC, Bijlenga D, et al. Induction of labour versus expectant management in women with preterm prelabour rupture of membranes between 34 and 37 weeks (the PPROMEXIL-trial). BMC Pregnancy Childbirth [Internet]. 2007;7(1):11. Available from: https://doi.org/10.1186/1471-2393-7-11

29. Van Der Heyden JL, Willekes C, Van Baar AL, Van Wassenaer-Leemhuis AG, Pajkrt E, Oudijk MA, et al. Behavioural and neurodevelopmental outcome of 2-year-old children after preterm premature rupture of membranes: Follow-up of a randomised clinical trial comparing induction of labour and expectant management. Eur J Obstet Gynecol Reprod Biol [Internet]. 2015 [cited 2021 May 27];194:17–23. Available from: https://www.sciencedirect.com/science/article/pii/S0301211515002511

30. van der Heyden J, Willekes C, Oudijk M, Porath M, Duvekot H (J), Bloemenkamp KW, et al. 712: Behavioral and developmental outcome of neonates at 2 years of age after preterm prelabor rupture of membranes: follow up of the PPROMEXIL trial. Am J Obstet Gynecol [Internet]. 2014 [cited 2021 May 27];210(1):S349–50. Available from: www.AJOG.org

31. Willekes C. Preterm premature rupture of membranes between 34 and 37 weeks: expectant management versus induction of labour (planned trial). [Internet]. 2007. Available from: Current Controlled Trials (www.controlled‐trials.com) (accessed 15 February 2007)

32. Vijgen SMC, Van Der Ham DP, Bijlenga D, Van Beek JJ, Bloemenkamp KWM, Kwee A, et al. Economic analysis comparing induction of labor and expectant management in women with preterm prelabor rupture of membranes between 34 and 37 weeks (PPROMEXIL trial). Acta Obstet Gynecol Scand [Internet]. 2014 Apr 1 [cited 2021 May 27];93(4):374–81. Available from: https://obgyn.onlinelibrary.wiley.com/doi/full/10.1111/aogs.12329

33. Van Der Ham DP, Van Der Heyden JL, Opmeer BC, Mulder ALM, Moonen RMJ, Van Beek JJ, et al. Management of late-preterm premature rupture of membranes: The PPROMEXIL-2 trial. Am J Obstet Gynecol. 2012 Oct 1;207(4):276.e1-276.e10.

34. Van der Ham D, Van der Heijden J, Opmeer B, Van Beek H, Willekes C, Mulder T, et al. Induction of labor versus expectant management in women with preterm prelabor rupture of membranes between 34 and 37 weeks ‐ the PPROMEXIL‐2 trial (ISRCTN05689407). Am J Obstet Gynecol. 2012;206(Suppl 1):S8‐9.

35. Kind P, Hardman G, Macran S. UK population norms for EQ-5D. Cent Heal Econ Univ York, Work Pap. 1999 Jan 1;

36. Pennington B, Wong R. Modelling carer health-related quality of life in NICE technology appraisals and highly specialised technologies. Sheffield; 2019.

37. National Institute for Health and Care Excellence. Strimvelis for treating adenosine deaminase deficiency–severe combined immunodeficiency. NICE. 2018.

38. Petrou S, Johnson S, Wolke D, Marlow N. The association between neurodevelopmental disability and economic outcomes during mid-childhood. Child Care Health Dev [Internet]. 2013 May [cited 2021 Mar 28];39(3):345–57. Available from: https://pubmed.ncbi.nlm.nih.gov/22372844/

39. Mujica-Mota RE, Roberts M, Abel G, Elliott M, Lyratzopoulos G, Roland M, et al. Common patterns of morbidity and multi-morbidity and their impact on health-related quality of life: evidence from a national survey. Qual Life Res [Internet]. 2015 Mar 20 [cited 2021 Mar 28];24(4):909–18. Available from: https://pubmed.ncbi.nlm.nih.gov/25344816/

40. Lain SJ, Roberts CL, Bond DM, Smith J, Morris JM. An economic evaluation of planned immediate versus delayed birth for preterm prelabour rupture of membranes: findings from the PPROMT randomised controlled trial. BJOG An Int J Obstet Gynaecol [Internet]. 2017 Mar 1;124(4):623–30. Available from: https://doi.org/10.1111/1471-0528.14302

41. NHS Improvement. 2016/17 reference costs and guidance [Internet]. NHS Improvement. 2017 [cited 2021 Apr 1]. Available from: https://webarchive.nationalarchives.gov.uk/20200501111106/https://improvement.nhs.uk/resources/reference-costs/

42. National Institute for Health and Care Excellence. Specialist neonatal respiratory care for babies born preterm. NICE. 2019.

43. Mangham LJ, Petrou S, Doyle LW, Draper ES, Marlow N. The cost of preterm birth throughout childhood in England and Wales. Pediatrics [Internet]. 2009 Feb [cited 2021 Mar 28];123(2). Available from: https://pubmed.ncbi.nlm.nih.gov/19171583/

44. Trotter CL, Edmunds w. J. Modelling cost effectiveness of meningococcal serogroup C conjugate vaccination campaign in England and Wales. BMJ [Internet]. 2002 Apr 6 [cited 2021 May 20];324(7341):809. Available from: www.doh.gov.uk/nhsexec/refcosts.htm

45. NHS Digital. Adult Social Care Activity and Finance Report, England - 2018-19 [PAS] - NHS Digital [Internet]. Adult Social Care Activity and Finance Report. 2019 [cited 2021 May 20]. Available from: https://digital.nhs.uk/data-and-information/publications/statistical/adult-social-care-activity-and-finance-report/2018-19

46. National Institute for Health and Care Excellence. Overview | Asthma: diagnosis, monitoring and chronic asthma management | Guidance | NICE [Internet]. NICE. 2017. Available from: https://www.nice.org.uk/guidance/ng80

47. Price DB, Saralaya D, Britton M, Thomas M, Haughney J, Pinnock H, et al. Asthma costs in the UK by asthma control status and exacerbations. In: A102 DOLLARS AND SENSE: IDENTIFYING THE COSTS, UTILIZATION AND BURDEN OF RESPIRATORY DISEASE MANAGEMENT. American Thoracic Society; 2013. p. A2145–A2145.

48. National Guideline Alliance. Twin pregnancy costing [Internet]. London; 2018. Available from: https://www.hfea.gov.uk/media/2650/nga-twin-pregnancy-costing-final.pdf

49. Curtis L, Burns A. Unit Costs of Health and Social Care 2019 [Internet]. Canterbury; 2019. Available from: https://kar.kent.ac.uk/79286/

50. National Institute for Health and Care Excellence. Overview | Intrapartum care for women with existing medical conditions or obstetric complications and their babies | Guidance | NICE [Internet]. NICE. 2019. Available from: https://www.nice.org.uk/guidance/ng121

51. Campbell HE, Kurinczuk JJ, Heazell AEP, Leal J, Rivero-Arias O. Healthcare and wider societal implications of stillbirth: a population-based cost-of-illness study. BJOG An Int J Obstet Gynaecol [Internet]. 2018 Jan 1 [cited 2021 May 20];125(2):108–17. Available from: https://pubmed.ncbi.nlm.nih.gov/29034559/
